# Plant macrofossil data for 48-0 ka in the USGS North American Packrat Midden Database, version 5.0

**DOI:** 10.1038/s41597-023-02616-y

**Published:** 2024-01-12

**Authors:** Laura E. Strickland, Robert S. Thompson, Sarah L. Shafer, Patrick J. Bartlein, Richard T. Pelltier, Katherine H. Anderson, R. Randall Schumann, Andrew K. McFadden

**Affiliations:** 1 U.S. Geological Survey, Geosciences and Environmental Change Science Center, Box 25046, Mail Stop 980, Denver Federal Center, Denver, Colorado 80225 USA; 2 U.S. Geological Survey, Geosciences and Environmental Change Science Center, 3200 SW Jefferson Way, Corvallis, Oregon 97331 USA; 3 Department of Geography, University of Oregon, Eugene, Oregon 97403-1251 USA; 4 Institute of Arctic and Alpine Research, University of Colorado, Boulder, Colorado 80309 USA; 5 Present Address: Abt Associates Inc., Tabor Center, 1200 17th St., 10th Floor, Denver, Colorado 80202 USA

**Keywords:** Palaeoclimate, Palaeoecology

## Abstract

Plant macrofossils from packrat (*Neotoma* spp.) middens provide direct evidence of past vegetation changes in arid regions of North America. Here we describe the newest version (version 5.0) of the U.S. Geological Survey (USGS) North American Packrat Midden Database. The database contains published and contributed data from 3,331 midden samples collected in southwest Canada, the western United States, and northern Mexico, with samples ranging in age from 48 ka to the present. The database includes original midden-sample macrofossil counts and relative-abundance data along with a standardized relative-abundance scheme that makes it easier to compare macrofossil data across midden-sample sites. In addition to the midden-sample data, this version of the midden database includes calibrated radiocarbon (^14^C) ages for the midden samples and plant functional type (PFT) assignments for the midden taxa. We also provide World Wildlife Fund ecoregion assignments and climate and bioclimate data for each midden-sample site location. The data are provided in tabular (.xlsx), comma-separated values (.csv), and relational database (.mdb) files.

## Background & Summary

In arid regions of North America, middens created by packrats (*Neotoma* spp.; also referred to as wood rats or trade rats) are an important source of paleobotanical information. Packrats collect plant debris and other material, typically from within a 50 m radius of their den sites, which are commonly located in caves and rockshelters^1^. The animals build middens near their dens by combining the collected materials with their fecal pellets and viscous urine and then compacting the mixture into voids and rock crevices. Over time, urine-cemented middens crystallize into solid masses preserving the plant remains inside. If middens are protected from weather, especially moisture, they may persist for tens of thousands of years after deposition^2–4^. Plant macrofossils (e.g., leaves, twigs, fruits, seeds) preserved in packrat middens often can be identified to the species level, providing inventories of plant communities found near a midden site in the past, although these inventories are biased by packrat foraging ranges and dietary preferences^5^. When radiocarbon dated, midden assemblages of various ages from multiple sites provide records of the spatial and temporal patterns of paleovegetation change over late Pleistocene and Holocene time periods^2,6–8^. In addition to providing records of vegetation change, packrat midden data have also been used for paleoclimate reconstructions^9^, for paleogenomic studies^10^, as a source of ancient DNA for investigating virus evolution^11^, to examine linkages between vegetation change and fluvial-system aggradation^12^, and for species-distribution modeling^13^.

One of the challenges in using paleobotanical midden data to investigate broad-scale patterns of climate and vegetation change is that a variety of different methods have been used to analyze plant macrofossil assemblages from middens and to classify the taxa they contain, making it difficult to compare and synthesize midden data. To address this issue, the U.S. Geological Survey (USGS) North American Packrat Midden Database was developed to provide researchers with access to standardized midden-derived paleobotanical data for investigating late Pleistocene and Holocene changes in plant species distributions in response to climate and environmental change. With support from the USGS and the National Oceanic and Atmospheric Administration (NOAA), the first online version of the midden database was released in 1998. In 2001, a data dictionary describing the structure of the 1998 database was published^14^, followed by an update to the online database in 2002. Version 3.0 of the database was released in 2006 and version 4.0 in 2016 with 3,205 packrat midden samples^15^. A timeline of database version releases, related publications, and conference presentations with abstracts are listed in Table 1.

**Table 1:** Timeline of midden database releases and related publications.

Database Version (Release Date)	Database Title, Authors, and Related Publications
Version 1.0 (1998)	USGS/NOAA North American Packrat Midden Database (R. R. Schumann, R. S. Thompson, K. H. Anderson). No longer available online.
	Related publication: USGS/NOAA North American Packrat Midden Database Data Dictionary (2001)^14^.
Version 2.0 (October 2002)	USGS/NOAA North American Packrat Midden Database (L. E. Strickland, R. R. Schumann, R. S. Thompson, K. H. Anderson). No longer available online.
	Related publication: Standardized paleobotanical data derived from packrat middens in western North America. Geological Society of America Annual Meeting abstract (October 2002)^89^.
Version 3.0 (October 2006)	USGS/NOAA North American Packrat Midden Database (L. E. Strickland, R. R. Schumann, R. S. Thompson, K. H. Anderson, R. T. Pelltier). No longer available online.
	Related publication: Quaternary plant fossils from caves and rockshelters: A database of paleoecological records from Neotoma middens in western North America. Geological Society of America Annual Meeting abstract (October 2006)^90^.
Version 4.0 (June 2016)	USGS/NOAA North American Packrat Midden Database (L. E. Strickland, R. R. Schumann, R. S. Thompson, K. H. Anderson, R. T. Pelltier)^15^. Currently available at: https://geochange.er.usgs.gov/midden/.
	Related publication: Quaternary plant fossils from *Neotoma* middens in western North America: An update of the USGS/NOAA North American Packrat Midden Database. American Quaternary Association (AMQUA) abstract (June 28-July 2, 2016; L. E. Strickland, R. R. Schumann, R. S. Thompson, K. H. Anderson, R. T. Pelltier).
Version 5.0 (October 2022)	USGS North American Packrat Midden Database (L. E. Strickland, R. S. Thompson, K. H. Anderson, R. T. Pelltier, S. L. Shafer, P. J. Bartlein, R. R. Schumann, A. K. McFadden)^16^. U.S. Geological Survey data release. Currently available at: 10.5066/P91UOARW.
	Related publication: Paleobotanical data from the USGS/NOAA North American Packrat Midden Database, version 5: Primary data, quality-assessed standardized data, and information on sample climatic and ecological context. Geological Society of America Annual Meeting abstract (September 2019)^91^.

**Fig. 1:**
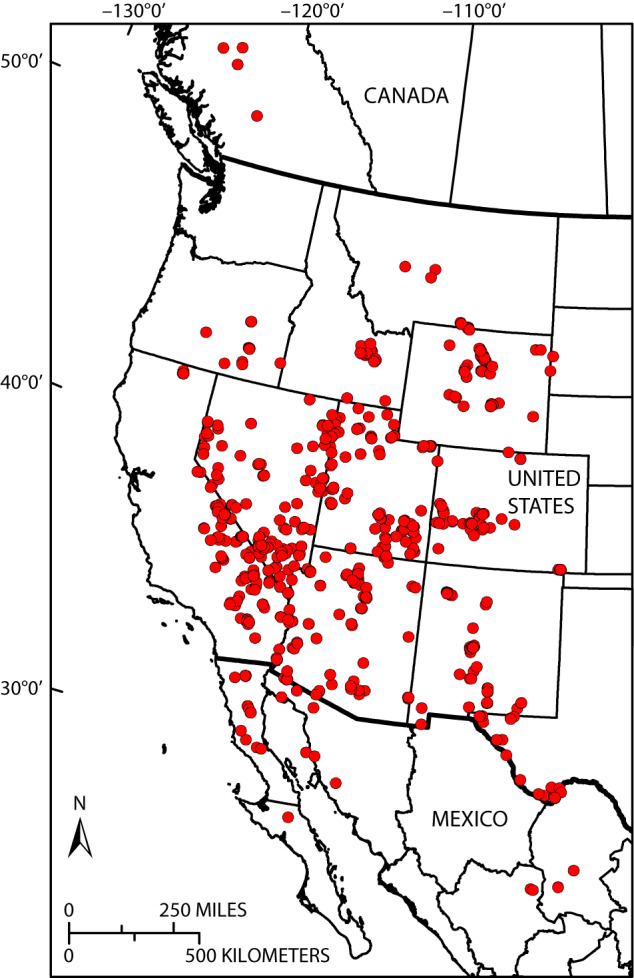
Locations (, red circles) of midden-sample sites included in the midden database. Figure from Strickland *et al*.^16^.

Here we describe version 5.0 of the USGS North American Packrat Midden Database with data available at 10.5066/P91UOARW^16^, including the methods used to compile the database and a description of the tables and variables included in the database. Version 5.0 contains original data from 3,331 packrat midden samples collected from permanently dry settings in caves and rock shelters in southwest Canada, the western United States, and northern Mexico (Fig. 1). Data, including location information, midden-sample ages, plant macrofossil taxon lists (from 2,336 midden samples), and taxon counts or relative-abundance data, were collected from published sources (e.g., journal articles, book chapters, theses, dissertations, government and private industry reports, and conference proceedings). Additional unpublished data were graciously contributed by researchers. The data were entered into a Microsoft Access relational database exactly as they were published or contributed without changing their original form unless noted. Taxon relative-abundance and count data were entered into the database with the intent to preserve the original sample count format. However, to facilitate the comparison and use of plant-macrofossil relative-abundance data from multiple sources, which are often published in a variety of formats, all relative-abundance and count data were also interpreted and translated into a simplified uniform-relative-abundance scheme. Compared to version 4.0, version 5.0 of the midden database has been expanded to include more precise midden-sample location data, calibration and evaluation of midden-sample age data, and assignment of plant functional types (PFTs) for the taxa in each midden sample. We have also assigned midden-sample sites to World Wildlife Fund (WWF) ecoregions and major habitat types (MHTs)^17^ and interpolated modern climate and bioclimate data^18,19^ to each midden-sample site location.

**Fig. 2:**
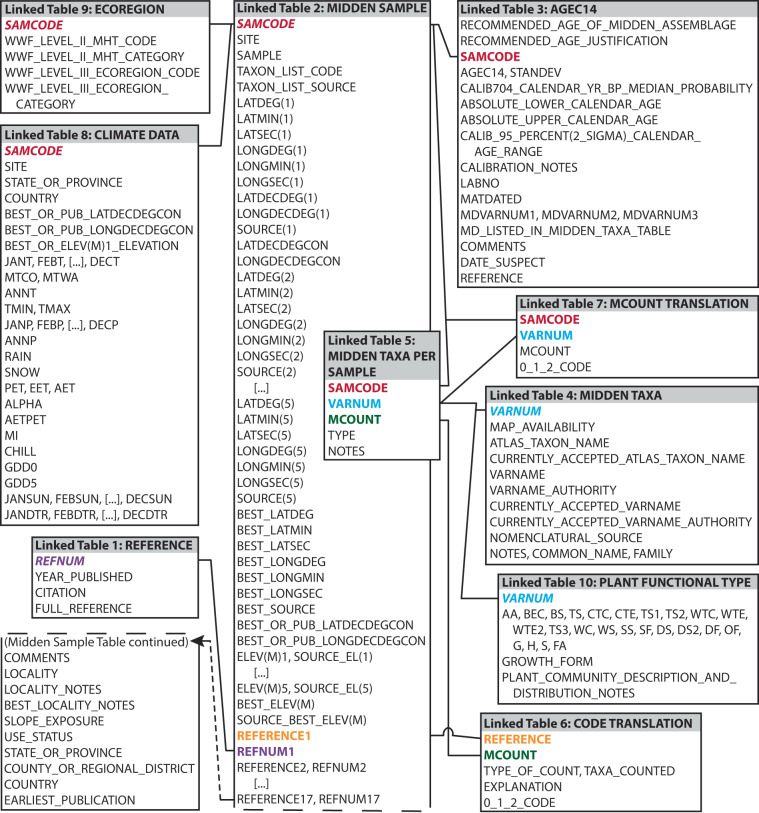
Relationships among the Linked data tables in the midden database. Each box lists the Linked data table name (bold black text), the common data fields in each table (bold red, blue, green, orange, and purple text) that are used to join the tables in the relational database, and the other data fields in each table (regular black text). Common data fields that provide unique identifiers for each table entry (i.e., each table row) are in italicized text. Note that the common data fields do not need to have the same name to be joined. For example, the REFERENCE field in the CODE TRANSLATION table (Linked Table 6) can be joined with the REFERENCE1 field in the MIDDEN SAMPLE table (Linked Table 2).

The USGS North American Packrat Midden Database (version 5.0) consists of 20 data tables arranged in a hierarchical fashion (listed in Tables 2–4)^16^. We refer to these data tables in the following text as “Linked” tables, “Unlinked” tables, and “Lookup” tables to distinguish them from the numbered tables in this paper (i.e., Tables 1–27). There are 10 Linked data tables in the Access relational database (see Table 2). Figure 2 displays the fields in each Linked data table and illustrates the relationships among the fields (these relationships may also be viewed within the relational database). Four Unlinked data tables (see Table 3) provide lists of: (1) synonymous taxon names (Unlinked Table 1) appearing in the Linked Table 4 MIDDEN TAXA table, (2) publications that contain midden-related information but did not contain midden-sample data suitable for inclusion in the database (Unlinked Table 2), (3) published abstracts related to packrat middens that did not contribute data to the database (Unlinked Table 3), (4) midden publications discussing middens located outside of North America that did not contribute data to the database (Unlinked Table 4). Six additional tables function as Lookup tables (see Table 4) which define: (1) use status codes indicating the completeness of the data provided for each midden sample (Lookup Table 1), (2) the 0-1-2 presence-absence relative-abundance codes (Lookup Table 2), (3) recommended age codes (Lookup Table 3), (4) taxon list codes (Lookup Table 4), (5) ecoregion codes (Lookup Table 5), and (6) PFT codes (Lookup Table 6).

**Table 2:** Linked data tables and descriptions of their content.

Linked Data Tables	Content
1. REFERENCE	List of data source publications.
2. MIDDEN SAMPLE	Sample codes and location information.
3. AGEC14	Radiocarbon ages, calibrated ages, and material dated.
4. MIDDEN TAXA	A comprehensive list of plant taxa recovered from midden samples included in this database.
5. MIDDEN TAXA PER SAMPLE	Lists plant taxon assemblages collected from each midden sample, original taxon-relative-abundance or count data, and organ type.
6. CODE TRANSLATION	Describes original macrofossil count data and macrofossil-relative-abundance schemes and explains how each count or scheme type was translated into a standard presence-absence scale.
7. MCOUNT TRANSLATION	Translates original macrofossil-relative-abundance or count data from the MIDDEN TAXA PER SAMPLE table (Linked Table 5) into a standard presence-absence scale (0-1-2 scale) using parameters defined in the CODE TRANSLATION table (Linked Table 6) and a make-table query.
8. CLIMATE DATA	Sixty-five climate and bioclimate variables, including monthly temperature and precipitation values interpolated to each midden-sample location.
9. ECOREGION	Midden-sample locations are assigned to one of 6 World Wildlife Fund (WWF) major habitat types (MHTs) and one of 24 WWF ecoregions^17^.
10. PLANT FUNCTIONAL TYPE	Midden taxa are assigned to 24 possible plant functional type (PFT) categories.

**Table 3:** Unlinked data tables and descriptions of their content.

Unlinked Data Tables	Content
1. SYNONYMS	Synonymous taxon names appearing in the MIDDEN TAXA table (Linked Table 4).
2. MIDDEN RELATED PUBLICATIONS WITH NO SIGNIFICANT DATA	Midden-related publications with no significant data content.
3. MIDDEN RELATED ABSTRACTS WITH NO SIGNIFICANT DATA	Midden-related abstracts with no significant data content.
4. MIDDEN PUBLICATIONS OUTSIDE NORTH AMERICA	Midden publications with midden sites outside of North America.

**Table 4:** Lookup data tables and descriptions of their content.

Lookup Data Tables	Content
1. USE STATUS CODE	A numeric scheme representing the completeness of available data used in the MIDDEN SAMPLE table (Linked Table 2).
2. 0-1-2 CODE	Defines the 0-1-2 presence-absence scale used in the CODE TRANSLATION and MCOUNT TRANSLATION tables (Linked Tables 6, 7).
3. RECOMMENDED AGE CODE	Defines symbols in the AGEC14 table (Linked Table 3) that indicate which age we consider best represents the midden sample/assemblage.
4. TAXON LIST CODE	Defines symbols in the MIDDEN SAMPLE table (Linked Table 2) that indicate when a taxon list is available and the completeness of the taxon list.
5. ECOREGION CODE	Defines abbreviations in the ECOREGION table (Linked Table 9) for the WWF MHTs and ecoregions^17^.
6. PLANT FUNCTIONAL TYPE CODE	Defines abbreviations in the PLANT FUNCTIONAL TYPE table (Linked Table 10) that represent the 24 plant functional type (PFT) categories.

**Table 5:** REFERENCE (Linked Table 1) field descriptions.

Column	Field Name	Description
A	REFNUM	Reference number. A unique number assigned to each source reference consisting of a number followed by the letter “m”.
B	YEAR_PUBLISHED	The year the source reference was published.
C	CITATION	Author(s) last name(s) and year of publication.
D	FULL_REFERENCE	Includes when available, the author(s) last name(s) and first initials, year of publication, title, journal or publication series, editor, publisher, page numbers, and URL.

Data release files: Linked_Table_1_REFERENCE.xlsx (.csv)^16^.

**Table 6:** MIDDEN SAMPLE (Linked Table 2) field descriptions.

Column	Field Name	Description
A	SAMCODE	Sample code. A unique code assigned to each midden sample.
B	SITE	Name of midden site.
C	SAMPLE	Original midden-sample identifier.
D	TAXON_LIST_CODE	A symbol in this field means a list of plant macrofossil taxa collected from the midden is available in the MIDDEN TAXA PER SAMPLE table (Linked Table 5; C = Complete taxon list, P = Partial taxon list, CU = Complete taxon list but unpublished and not available, PU = Partial taxon list but unpublished and not available). Codes are also defined in the TAXON LIST CODE table (Lookup Table 4).
E	TAXON_LIST_SOURCE	Source publication REFNUM(s) for the plant macrofossil taxon list.
F	LATDEG(1)	Published degrees of north latitude (set 1).
G	LATMIN(1)	Published minutes of north latitude (set 1).
H	LATSEC(1)	Published seconds of north latitude (set 1).
I	LONGDEG(1)	Published degrees of west longitude (set 1).
J	LONGMIN(1)	Published minutes of west longitude (set 1).
K	LONGSEC(1)	Published seconds of west longitude (set 1).
L	LATDECDEG(1)	Latitude published in decimal degrees, sometimes used to convert to degrees, minutes, seconds and populate latitude (set 1) fields.
M	LONGDECDEG(1)	Longitude published in decimal degrees, sometimes used to convert to degrees, minutes, seconds and populate longitude (set 1) fields.
N	SOURCE(1)	Latitude and longitude (set 1) data source REFNUM.
O	LATDECDEGCON	Latitude degrees, minutes, seconds (set 1) converted to decimal degrees.
P	LONGDECDEGCON	Longitude degrees, minutes, seconds (set 1) converted to decimal degrees.
Q-AR	LATDEG(2–5)	Degrees of north latitude (sets 2–5).
Q-AR	LATMIN(2–5)	Minutes of north latitude (sets 2–5).
Q-AR	LATSEC(2–5)	Seconds of north latitude (sets 2–5).
Q-AR	LONGDEG(2–5)	Degrees of west longitude (sets 2–5).
Q-AR	LONGMIN(2–5)	Minutes of west longitude (sets 2–5).
Q-AR	LONGSEC(2–5)	Seconds of west longitude (sets 2–5).
Q-AR	SOURCE(2–5)	Latitude and longitude (sets 2–5) data source REFNUM.
AS	BEST_LATDEG	Improved degrees of north latitude estimate.
AT	BEST_LATMIN	Improved minutes of north latitude estimate.
AU	BEST_LATSEC	Improved seconds of north latitude estimate.
AV	BEST_LONGDEG	Improved degrees of west longitude estimate.
AW	BEST_LONGMIN	Improved minutes of west longitude estimate.
AX	BEST_LONGSEC	Improved seconds of west longitude estimate.
AY	BEST_SOURCE	Sources utilized to estimate best location coordinates.
AZ	BEST_OR_PUB_LATDECDEGCON	Degrees, minutes, seconds of latitude converted to decimal degrees using “BEST_” coordinates when available or published coordinates if “BEST_” coordinates are not provided.
BA	BEST_OR_PUB_LONGDECDEGCON	Degrees, minutes, seconds of longitude converted to decimal degrees using “BEST_” coordinates when available or published coordinates if “BEST_” coordinates are not provided.
BB	ELEV(M)1	Elevation (value 1) in meters.
BC	SOURCE_EL(1)	Elevation data source (value 1) REFNUM.
BD-BK	ELEV(M)2–5	Elevation (values 2–5) in meters.
BD-BK	SOURCE_EL(2–5)	Elevation data source (values 2–5) REFNUM.
BL	BEST_ELEV(M)	Improved elevation estimate, in meters.
BM	SOURCE_BEST_ELEV(M)	Sources utilized to estimate best elevation.
BN	REFERENCE1	Author and year of the primary reference, typically the earliest publication and usually includes the taxon list.
BO	REFNUM1	Reference number of primary reference (REFERENCE1).
BP-CU	REFERENCE2-17	Additional publications containing new or repeated publication of data pertaining to the midden.
BP-CU	REFNUM2-17	Reference numbers of secondary references (REFERENCE2 to REFERENCE17).
CV	COMMENTS	Miscellaneous comments.
CW	LOCALITY	General geographic region where midden sample was collected (e.g., mountain range or stream valley).
CX	LOCALITY_NOTES	Detailed information about the midden-sample collection location.
CY	BEST_LOCALITY_NOTES	Description of how the best location data were determined and how the original location data were altered.
CZ	SLOPE_EXPOSURE	Geographic direction midden faces (N = north, S = south, E = east, W = west).
DA	USE_STATUS	Describes the completeness of the data available using a numeric code which is defined below and in the USE STATUS CODE table [Lookup Table 1; 1 = Sample information, age data, complete taxon list published, 2 = Sample information and age data, no taxon list, 3 = Sample information and partial or complete taxon list, no age data, 4 = Sample information, no taxon list or age data (data may be unpublished), −9 = Sample information, age data, and partial taxon list published].
DB	STATE_OR_PROVINCE	State name for sites in the United States of America (USA) and Mexico, or province name for sites in Canada.
DC	COUNTY_OR_REGIONAL_DISTRICT	County name for sites in the USA or regional district name for sites in Canada.
DD	COUNTRY	Country name (USA, Mexico, or Canada).
DE	EARLIEST_PUBLICATION	Year of earliest publication.

Data release files: Linked_Table_2_MIDDEN_SAMPLE.xlsx (.csv)^16^.

**Table 7:** AGEC14 (Linked Table 3) field descriptions.

Column	Field Name	Description
A	RECOMMENDED_AGE_OF _ MIDDEN_ASSEMBLAGE	Symbols indicate the preferred age for representing the midden sample/assemblage. Symbols are defined below and in the RECOMMENDED AGE CODE table (Lookup Table 3). X = Recommended age to represent the midden sample/assemblage. X1-PA or X1-PA(FST) = Pooled Age (PA) or Pooled Age (Failed Significance Test). Primary preferred age when there are multiple ages on a sample. X2 = Secondary preferred age when there are multiple ages on a sample. Age with the smaller standard deviation. X? = Recommended age to represent the midden sample/assemblage but use with caution. ? = Multiple ages are not within two standard deviations of each other, and it is uncertain which age represents the midden sample/assemblage.
B	RECOMMENDED_AGE_JUSTIFICATION	Reasoning for age recommendation.
C	SAMCODE	Sample code. A unique code assigned to each midden sample.
D	AGEC14	Radiocarbon age in years before present.
E	STANDEV	Standard deviation (measure of analytical uncertainty).
F	CALIB704_CALENDAR_YR_BP_MEDIAN_PROBABILITY	Calibrated radiocarbon calendar age median probability generated using CALIB (rev. 7.0.4).
G	ABSOLUTE_LOWER_CALENDAR_AGE	2 sigma absolute youngest probable calendar age.
H	ABSOLUTE_UPPER_CALENDAR_AGE	2 sigma absolute oldest probable calendar age.
I	CALIB_95_PERCENT(2_SIGMA)_CALENDAR_AGE_RANGE	2 sigma probable calendar age range.
J	CALIBRATION_NOTES	Comments related to radiocarbon age calibration.
K	LABNO	A sample identification number assigned by the radiocarbon laboratory.
L	MATDATED	Material dated. May consist of various plant materials, dung pellets, or midden matrix. If the age is an average of multiple ages, the material used for each age is separated by a slash.
M	MDVARNUM1	Material dated variable number (corresponds to the VARNUM values in Linked Table 4), a unique number representing the first plant taxon dated in the sample.
N	MDVARNUM2	Material dated variable number (corresponds to the VARNUM values in Linked Table 4), a unique number representing the second plant taxon dated in the sample.
O	MDVARNUM3	Material dated variable number (corresponds to the VARNUM values in Linked Table 4), a unique number representing the third plant taxon dated in the sample.
P	MD_LISTED_IN_MIDDEN_TAXA_TABLE	Indicates whether the material dated is included in the midden taxon assemblage list.
Q	COMMENTS	Miscellaneous comments.
R	DATE_SUSPECT	An “X” indicates that the age may be in error, or the material dated may be an older or younger contaminant.
S	REFERENCE	Published source(s) for the age data.

Data release files: Linked_Table_3_AGEC14.xlsx (.csv)^16^.

**Table 8:** MIDDEN TAXA (Linked Table 4) field descriptions.

Column	Field Name	Description
A	VARNUM	Variable number. A unique number assigned to each midden taxon.
B	MAP_AVAILABILITY	A “1” indicates that a modern geographic distribution map for the midden taxon is available in the Thompson *et al*. Atlas^92,93^. A “0” indicates a map is not available in the Atlas. We did not indicate distribution map availability for midden taxa without species level identifications or for taxa with some degree of uncertainty in the species identification such as names containing “cf.” or “-type”. It is likely that midden taxa identified at the variety or subspecies level are included in species-level map coverages; however, we only list map coverage availability in association with species-level midden taxa and not for individual varieties or subspecies. Digital versions of species distribution maps from Thompson *et al*.^92^ can be viewed online at 10.3133/pp1650G.
C	ATLAS_TAXON_NAME	Name of the Atlas taxon distribution map^92,93^ that best represents the midden taxon. This name comes from the original mapped distribution source publication; therefore, the Atlas taxon name may not be the currently accepted name of the taxon.
D	CURRENTLY_ACCEPTED_ATLAS_TAXON_NAME	Currently accepted name of the mapped Atlas taxon^92,93^. This name is provided when the original map name has changed or when the original map distribution represents different taxa than the original name suggests.
E	VARNAME	Variable name. The original Latin botanical name and syntax for each taxon as used by the midden macrofossil analyst.
F	VARNAME_AUTHORITY	The name(s) identifying the person(s) who described and validly published the plant taxon name. Should the original taxon name have been revised, the name of the original authority is shown in parentheses followed by the name of the authority who created the existing taxon name. The names of authorities are abbreviated.
G	CURRENTLY_ACCEPTED_VARNAME	The currently accepted Latin botanical name for the taxon (“same” indicates that the currently accepted taxon name is the same as the VARNAME).
H	CURRENTLY_ACCEPTED_VARNAME_AUTHORITY	The name(s) identifying the person(s) who assigned the original taxon name (in parentheses) followed by the authority of the currently accepted taxon name who is responsible for defining the taxonomic name change (“same” indicates that the currently accepted authority is the same as the VARNAME_AUTHORITY).
I	NOMENCLATURAL_SOURCE	Source reference for nomenclatural information.
J	NOTES	Miscellaneous notes.
K	COMMON_NAME	Common name(s).
L	FAMILY	Latin name of the family of plants to which the taxon belongs.

Data release files: Linked_Table_4_MIDDEN_TAXA.xlsx (.csv)^16^.

**Table 9:** MIDDEN TAXA PER SAMPLE (Linked Table 5) field descriptions.

Column	Field Name	Description
A	SAMCODE	Sample code. A unique code assigned to each midden sample.
B	VARNUM	Variable number. A unique number assigned to each midden taxon.
C	MCOUNT	Macrofossil count. Original symbols or numbers used to represent plant macrofossil relative abundance or counts, defined in the CODE TRANSLATION table (Linked Table 6).
D	TYPE	Plant organs identified (needle, seed, leaf, etc.).
E	NOTES	Miscellaneous notes.

Data release files: Linked_Table_5_MIDDEN_TAXA_PER_SAMPLE.xlsx (.csv)^16^.

**Table 10:** CODE TRANSLATION (Linked Table 6) field descriptions.

Column	Field Name	Description
A	REFERENCE	Citation including author(s) and date of publication of taxon list source publication.
B	MCOUNT	Macrofossil count. Original symbols or numbers used to represent plant macrofossil relative abundance or counts, defined in the EXPLANATION field.
C	TYPE_OF_COUNT	General type of counting scheme used.
D	TAXA_COUNTED	Specifies whether the publication provides complete or partial taxon lists or if the taxon was added from material dated information.
E	EXPLANATION	Defines the symbols used to represent relative abundance in the source publication.
F	0_1_2_CODE	The original MCOUNT value or symbol translated into a standard 0-1-2 presence-absence scale (0 = Absent, 1 = Rare, 2 = Present, 7 = Cannot translate, 9 = Possible contaminant).

Data release files: Linked_Table_6_CODE_TRANSLATION.xlsx (.csv)^16^.

**Table 11:** MCOUNT TRANSLATION (Linked Table 7) field descriptions.

Column	Field Name	Description
A	SAMCODE	Sample code. A unique code assigned to each midden sample.
B	VARNUM	Variable number. A unique number assigned to each midden taxon.
C	MCOUNT	Macrofossil count. Original symbols or numbers used to represent plant macrofossil relative abundance or counts, defined in the CODE TRANSLATION table (Linked Table 6).
D	0_1_2_CODE	The original macrofossil relative abundance or counts translated into a standard 0-1-2 presence-absence scale.

Data release files: Linked_Table_7_MCOUNT_TRANSLATION.xlsx (.csv)^16^.

**Table 12:** CLIMATE DATA (Linked Table 8) field descriptions.

Column	Field Name	Description
A	SAMCODE	Sample code. A unique code assigned to each midden sample.
B	SITE	Name of midden site.
C	STATE_OR_PROVINCE	State name for sites in the USA and Mexico, or province name for sites in Canada.
D	COUNTRY	Country name (USA, Mexico, or Canada).
E	BEST_OR_PUB_LATDECDEGCON	Latitude (decimal degrees) used for climate interpolation. These latitude values are from the MIDDEN SAMPLE table (Linked Table 2).
F	BEST_OR_PUB_LONGDECDEGCON	Longitude (decimal degrees) used for climate interpolation. These longitude values are from the MIDDEN SAMPLE table (Linked Table 2).
G	BEST_OR_ELEV(M)1_ELEVATION	Elevation (meters) used for climate interpolation.
H-S	JANT – DECT	January to December monthly mean temperature (°C; 1961–1990 30-year mean).
T	MTCO^a^	Mean temperature of the coldest month (°C; 1961–1990 30-year mean).
U	MTWA^a^	Mean temperature of the warmest month (°C; 1961–1990 30-year mean).
V	ANNT^a^	Mean annual temperature (°C; 1961–1990 30-year mean).
W	TMIN	Absolute minimum temperature (°C; 1951–1980).
X	TMAX	Absolute maximum temperature (°C; 1951–1980).
Y-AJ	JANP – DECP	January to December monthly mean total precipitation (mm; 1961–1990 30-year mean).
AK	ANNP^a^	Mean annual total precipitation (mm; 1961–1990 30-year mean).
AL	RAIN^a^	Rain (mm) component of mean annual total precipitation.
AM	SNOW^a^	Snowfall snow water equivalent (mm) component of mean annual total precipitation.
AN	PET^a^	Mean annual potential evapotranspiration (mm).
AO	EET^a^	Mean annual equilibrium evapotranspiration (mm).
AP	AET^a^	Mean annual actual evapotranspiration (mm).
AQ	ALPHA^a^	Annual Priestley-Taylor coefficient (α) calculated as AET divided by EET (dimensionless)^94^.
AR	AETPET	Annual evaporation ratio calculated as AET divided by PET (dimensionless).
AS	MI	Annual moisture index calculated as ANNP divided by PET (dimensionless).
AT	CHILL^a^	Chilling period (number of days with temperature < 5 °C).
AU	GDD0^a^	Growing-degree days on a 0 °C base.
AV	GDD5^a^	Growing-degree days on a 5 °C base.
AW-BH	JANSUN – DECSUN	January to December mean monthly maximum possible sunshine (% of daylength; 1961–1990 30-year mean).
BI-BT	JANDTR – DECDTR	January to December mean monthly diurnal temperature range (°C; 1961–1990 30-year mean).

Data release files: Linked_Table_8_CLIMATE_DATA.xlsx (.csv)^16^.

^a^Variable calculated using SPLASH^19^ (SPLASH code is available from https://bitbucket.org/labprentice/splash/src/master/).

**Table 13:** WWF ecoregion classification for packrat midden sites.

Level II Major Habitat Type	Level III Ecoregion
Grasslands/Savanna/Shrub (GSS)	Montana Valley and Foothill Grasslands (MVFG) Northern Short Grasslands (NSG) Western Short Grasslands (WSG)
Temperate Coniferous Forests (TCF)	Arizona Mountains Forests (AMF) Blue Mountains Forest (BMF) Cascade Mountains Leeward Forest (CMLF) Colorado Rockies Forests (CRF) Eastern Cascades Forest (ECF) Fraser Plateau and Basin complex Forests (FPBF) Great Basin Montane Forests (GBMF) Sierra Juarez and San Pedro Martir Pine Oak Forest (SSPOF) Sierra Nevada Forests (SNF) South Central Rockies Forests (SCRF) Wasatch Uinta Montane Forests (WUMF)
Mediterranean Scrub and Savanna (MSS)	California Coastal Sage and Chaparral (CCSC).
Xeric Shrublands/Scrublands (XSS)	Snake/Columbia Shrub Steppe (SCSS) Wyoming Basin Shrub Steppe (WBSS) Great Basin Shrub Steppe (GBSS) Colorado Plateau Shrubland (CPS)
Xeric Deserts (XD)	Baja California Desert (BCD) Chihuahuan Desert (CD) Mojave Desert (MD) Sonoran Desert (SD)
Broadleaf and Mixed Forests (BMF)	Sierra Madre Occidental Pine Oak Forest (SMPOF)

**Table 14:** ECOREGION (Linked Table 9) field descriptions.

Column	Field Name	Description
A	SAMCODE	Sample code. A unique code assigned to each midden sample.
B	WWF_LEVEL_II_MHT_CODE	Two- or three-letter code representing 1 of 6 possible level II MHT categories.
C	WWF_LEVEL_II_MHT_CATEGORY	Level II MHT category definition.
D	WWF_LEVEL_III_ECOREGION_CODE	Two-, three-, four-, or five-letter code representing 1 of 24 possible level III ecoregion categories.
E	WWF_LEVEL_III_ECOREGION_CATEGORY	Level III ecoregion category definition.

Data release files: Linked_Table_9_ECOREGION.xlsx (.csv)^16^.

**Table 15:** PLANT FUNCTIONAL TYPE (Linked Table 10) field descriptions.

Column	Field Name	Description
A	VARNUM	Variable number. A unique number assigned to each midden taxon.
B	AA	Arctic/Alpine
C	BEC	Boreal Evergreen Conifer
D	BS	Boreal Summergreen
E	TS	Temperate Summergreen
F	CTC	Cool Temperate Conifer
G	CTE	Cool Temperate broadleaved Evergreen
H	TS1	cool Temperate Summergreen
I	TS2	intermediate Temperate Summergreen
J	WTC	Warm Temperate Conifer
K	WTE	Warm Temperate broadleaved Evergreen
L	WTE2	Warm Temperate broadleaved Evergreen sclerophyll
M	TS3	warm Temperate Summergreen
N	WC	Woodland Conifer
O	WS	Woodland broadleaved tree/Shrub
P	SS	Steppe tree/Shrub
Q	SF	Steppe Forb
R	DS	Desert tree/Shrub
S	DS2	Desert tree/Shrub frost sensitive
T	DF	Desert Forb
U	OF	Other Forb
V	G	Grass
W	H	Heath
X	S	Sedge
Y	FA	Ferns and Fern Allies
Z	GROWTH_FORM	Categorizes each taxon by growth form. If a taxon belongs to more than one category (for example, trees and shrubs) we assigned the taxon to the lowest category (tree). 0 = tree, 5 = shrub (includes subshrubs), 8 = forbs and grasses.
AA	PLANT_COMMUNITY_DESCRIPTION_AND_DISTRIBUTION_NOTES	Notes collected from various floras describing the geographic occurrence of the taxon and the plant communities in which the taxon is commonly found.

Data release files: Linked_Table_10_PLANT_FUNCTIONAL_TYPE.xlsx (.csv)^16^.

**Table 16:** Online resources consulted for plant functional type (PFT) evaluation and classification (accessed 2011–2020).

Online Resource	URL
Calflora, information on wild California plants.	https://www.calflora.org/
California Native Plant Society, CALSCAPE.	https://calscape.org/
Fire Effects Information System (FEIS). Fire effects information system (FEIS) online database, U.S. Department of Agriculture Forest Service, Rocky Mountain Research Station, Fire Sciences Laboratory.	https://feis-crs.org/feis/
Flora of North America, eFloras: St. Louis, MO, Missouri Botanical Garden, and Cambridge, MA, Harvard University Herbaria.	http://www.efloras.org
Southwest Environmental Information Network (SEINet). SEINet Data Portal.	https://swbiodiversity.org/seinet/
Texas Native Plants Database (Benny Simpson’s Texas native trees).	https://aggie-horticulture.tamu.edu/ornamentals/natives/tamuhort.html
The Biota of North America Program, BONAP.	http://bonap.net/napa
The Jepson Herbarium eFlora, University of California, Berkeley.	http://ucjeps.berkeley.edu/eflora/
U.S. Department of Agriculture Natural Resources Conservation Service (USDA NRCS). The PLANTS Database, National Plant Data Team, Greensboro, NC, USA.	https://plants.sc.egov.usda.gov/ home

**Table 17:** Plant functional type (PFT) category descriptions and representative taxa.

Climatic Zone or Biome	PFTs and Representative Taxa
ARCTIC	**AA** = Arctic/Alpine dwarf shrubs and perennial herbs: needleleaved, scale-leaved, or broadleaved; deciduous or evergreen. Includes midden database taxa: *Juniperus communis*, *J. horizontalis*, *Phlox* sp., and Polemoniaceae. **OF** = Other Forbs, arctic forbs are placed in this category.
BOREAL	**BEC** = Boreal Evergreen Conifer trees/shrubs, needleleaved and scale-leaved. Includes midden database taxa: *Juniperus communis*, *J. horizontalis, Pseudotsuga menziesii*, Pinaceae*, Picea* sp., and *Pinus* sp. **BS** = Boreal Summergreen trees/shrubs, broadleaved. Includes midden database taxa: *Alnus* spp., *Amelanchier alnifolia*, *Betula papyrifera*-type, *Ceanothus* sp., *Cornus* spp., *Fraxinus* sp., *Populus* sp., *Dasiphora fruticosa*, *Prunus* sp., *Rhamnus* sp., *Rhus* sp., *Ribes montigenum, Rosa woodsii, Rubus* sp.*, Salix lasiandra, Sambucus* spp*., Shepherdia canadensis*, and *Symphoricarpos* spp. **OF** = Other Forbs, boreal forbs are placed in this category.
TEMPERATE	**TS** = General category for Temperate Summergreen trees/shrubs (includes **TS1,** **TS2**, and **TS3** categories). Taxa assigned to the TS category are not always differentiated into **TS1,** **TS2**, or **TS3** categories.
Cool Temperate	**CTC** = Cool Temperate Conifer trees/shrubs, evergreen. Includes midden database taxa: *Abies* spp., *Juniperus* spp., *Picea* spp., and *Pinus* spp. **CTE** = Cool Temperate broadleaved Evergreen trees/shrubs, includes succulents (*Agave* and Cactaceae). Includes midden database genera: *Arctostaphylos, Artemisia, Berberis, Cercocarpus, Ericameria, Purshia, Quercus, Rhamnus*, and *Yucca*. **TS1** = cool Temperate Summergreen trees/shrubs. Includes midden database genera: *Acer, Amelanchier, Artemisia, Betula, Celtis, Populus, Prunus, Purshia, Quercus, Rhus, Ribes, Salix, Sambucus*, and *Symphoricarpos*.
Intermediate Temperate	**TS2** = intermediate Temperate Summergreen trees/shrubs.
Warm Temperate	Includes plants occurring in Mediterranean type chaparral, coastal sage scrub, and oak woodland within California (USA), in chaparral and oak woodland within Arizona (USA) and New Mexico (USA), and oak woodland within Texas (USA). **WTC** = Warm Temperate Conifer trees/shrubs, evergreen. Includes midden database taxa: *Abies concolor*, *Calocedrus decurrens*, *Hesperocyparis* spp., *Pinus discolor*, *Pinus monophylla*, and *Torreya californica*. **WTE** = Warm Temperate broadleaved Evergreen trees/shrubs, also semi-evergreen (drought deciduous) plants, includes succulents (*Agave* and Cactaceae). Includes midden database genera and families: *Arctostaphylos*, Cactaceae, *Cercocarpus, Ephedra, Ericameria*, *Quercus, Rhamnus*, and *Yucca*. **WTE2** = Warm Temperate broadleaved Evergreen sclerophyll trees/shrubs, plants with hard, leathery, evergreen foliage. Includes midden database genera: *Arctostaphylos*, *Baccharis, Ceanothus, Quercus*, and *Vauquelinia*. **TS3** = warm Temperate Summergreen trees/shrubs. Includes midden database genera: *Bursera, Encelia, Eriogonum, Lycium, Nolina, Populus, Prosopis, Prunus, Rhus*, and *Sambucus*. **OF** = Other Forbs, temperate forbs are placed in this category.
OPEN CONIFER WOODLAND (PINYON – JUNIPER WOODLAND)	**WC** = Woodland Conifer trees/shrubs, evergreen, needleleaved and scale-leaved. Includes midden database taxa: *Hesperocyparis* spp., *Juniperus ashei, J. communis*, *J. deppeana, J. horizontalis, J. osteosperma, J. pinchotii*, *J. scopulorum*, *Pinus cembroides, P. edulis*, *P. flexilis, P. monophylla*, *P. quadrifolia*, and *P. remota*. **WS** = Woodland broadleaved trees/Shrubs, evergreen or summergreen, including succulents (Agavaceae and Cactaceae). Includes midden database genera: *Agave, Artemisia, Atriplex, Brickellia, Echinocereus, Ephedra*, *Ericameria, Eriogonum, Forsellesia, Gutierrezia, Holodiscus, Lycium, Mortonia, Opuntia, Prunus, Psorothamnus, Quercus, Rosa, Salvia, Sclerocactus, Shepherdia, Symphoricarpos, Tetradymia*, and *Yucca*. **OF** = Other Forbs, conifer woodland forbs are placed in this category.
STEPPE	**SS** = Steppe trees/Shrubs, broadleaved (evergreen or summergreen). Includes subshrubs or suffrutescent shrubs with woody bases, and succulents (*Agave* and Cactaceae). Includes midden database genera and families: *Agave*, *Amelanchier*, *Artemisia*, *Atriplex*, *Berberis*, *Brickellia*, Cactaceae, *Cercocarpus*, *Ephedra*, *Ericameria*, *Gutierrezia*, *Potentilla*, *Prosopis*, *Prunus*, *Purshia*, *Quercus*, *Rhus*, *Ribes*, *Salvia*, *Sambucus*, *Tetradymia*, and *Yucca*. **SF** = Steppe herbaceous Forbs. Found in grassland or prairie environments.
DESERT	**DS** = Desert trees/Shrubs, broadleaved (evergreen or summergreen), plants have a strategy for survival in desert climates such as stem photosynthesis, drought deciduousness, and succulence. Includes subshrubs or suffrutescent shrubs with woody bases, and succulents (*Agave* and Cactaceae). Includes midden database taxa: *Agave*, *Ambrosia*, *Artemisia*, *Atriplex*, *Berberis*, *Brickellia*, *Buddleja*, Cactaceae, *Cercidium*, *Condalia*, *Dasylirion wheeleri*, *Ephedra*, *Fallugia paradoxa*, *Fouquieria*, *Fraxinus*, *Ericameria*, *Gutierrezia*, *Jatropha*, *Larrea tridentata*, *Mortonia*, *Olneya*, *Parkinsonia*, *Prosopis*, *Psorothamnus*, *Rhus*, *Sarcobatus*, *Senegalia*, *Vachellia*, *Viguiera*, and *Yucca*. **DS2** = Desert trees/Shrubs, frost sensitive (not annuals or perennials that regenerate after frost). Includes midden database taxa: *Bursera microphylla*, *Cereus giganteus*, *Olneya tesota*, *Pachycereus schottii*, *Parkinsonia*, and *Stenocereus thurberi*. **DF** = Desert herbaceous Forbs.
OTHER	**G** = Grass, all Poaceae. **S** = Sedge, all Cyperaceae. **H** = Heath, all Ericaceae. **FA** = Ferns and Fern Allies, non-vascular bryophytes (liverwort, hornwort, true moss) and seedless vascular plants: Lycophyta (club mosses), Psilotophyta (*Psilotum*), Pterophyta (ferns), and Sphenophyta (horsetails). **OF** = Other Forbs, includes weedy species, all forbs other than desert and steppe forbs such as arctic, boreal, temperate, and open conifer woodland forbs.

**Table 18:** SYNONYMS (Unlinked Table 1) field descriptions.

Column	Field Name	Description
A	VARNUM	Variable number. A unique number assigned to each midden taxon.
B	VARNAME	Variable name. The original Latin botanical name and syntax used by the midden macrofossil analyst.
C-O	VARNUM2-8	Variable numbers 2–8.
D-P	SYNONYM_VARNAME2–8	Synonymous variable names 2–8.
Q	CURRENTLY_ACCEPTED_VARNAME	The currently accepted Latin botanical name.
R	NOTES	Note indicates when an autonym is the accepted name.

Data release files: Unlinked_Table_1_SYNONYMS.xls (.csv)^16^.

**Table 19:** MIDDEN RELATED PUBLICATIONS WITH NO SIGNIFICANT DATA (Unlinked Table 2) field descriptions.

Column	Field Name	Description
A	YEAR_PUBLISHED	The year the source reference was published.
B	CITATION	Author(s) last name(s) and year of publication.
C	FULL_REFERENCE	Includes when available, the author(s) last name(s) and first initials, year of publication, title, journal or publication series, editor, publisher, page numbers, and URL.

Data release files: Unlinked_Table_2_MIDDEN_RELATED_PUBLICATIONS_WITH_NO_SIGNIFICANT_DATA.xlsx (.csv)^16^.

**Table 20:** MIDDEN RELATED ABSTRACTS WITH NO SIGNIFICANT DATA (Unlinked Table 3) field descriptions.

Column	Field Name	Description
A	YEAR_PUBLISHED	The year the source reference was published.
B	CITATION	Author(s) last name(s) and year of publication.
C	FULL_REFERENCE	Includes when available, the author(s) last name(s) and first initials, year of publication, title, journal, program or abstract volume, page numbers, and URL.

Data release files: Unlinked_Table_3_MIDDEN_RELATED_ABSTRACTS_WITH_NO_SIGNIFICANT_DATA.xlsx (.csv)^16^.

**Table 21:** MIDDEN PUBLICATIONS OUTSIDE NORTH AMERICA (Unlinked Table 4) field descriptions.

Column	Field Name	Description
A	YEAR_PUBLISHED	The year the source reference was published.
B	CITATION	Author(s) last name(s) and year of publication.
C	FULL_REFERENCE	Includes when available, the author(s) last name(s) and first initials, year of publication, title, journal or publication series, editor, publisher, page numbers, and URL.

Data release files: Unlinked_Table_4_MIDDEN_PUBLICATIONS_OUTSIDE_NORTH_AMERICA.xlsx (.csv)^16^.

**Table 22:** USE STATUS CODE (Lookup Table 1) field descriptions.

Column	Field Name	Description
A	USE_STATUS_CODE	Status codes: −9, 1, 2, 3, 4.
B	CODE_DEFINITION	Definition for each use status code.

Data release files: Lookup_Table_1_USE_STATUS_CODE.xlsx (.csv)^16^.

**Table 23:** 0-1-2 CODE (Lookup Table 2) field descriptions.

Column	Field Name	Description
A	0_1_2_CODE	Codes: 0, 1, 2, 7, 9.
B	CODE_DEFINITION	Definition for each 0-1-2 code.

Data release files: Lookup_Table_2_0_1_2_CODE.xlsx (.csv)^16^.

**Table 24:** RECOMMENDED AGE CODE (Lookup Table 3) field descriptions.

Column	Field Name	Description
A	AGE_CODE	Age codes: X, X?, X1-PA, X1-PA(FST), X2, ?.
B	CODE_DEFINITION	Definition for each age code.

Data release files: Lookup_Table_3_RECOMMENDED_AGE_CODE.xlsx (.csv)^16^.

**Table 25:** TAXON LIST CODE (Lookup Table 4) field descriptions.

Column	Field Name	Description
A	TAXON_LIST_CODE	Taxon list codes: C, CU, P, PU.
B	CODE_DEFINITION	Definition for each taxon list code.

Data release files: Lookup_Table_4_TAXON_LIST_CODE.xlsx (.csv)^16^.

**Table 26:** ECOREGION CODE (Lookup Table 5) field descriptions.

Column	Field Name	Description
A	WWF_LEVEL_III_ECOREGION_CODE	WWF level III ecoregion codes.
B	WWF_LEVEL_III_ECOREGION_CATEGORY	WWF level III ecoregion names.
C	WWF_LEVEL_II_MHT_CODE	WWF level II major habitat type (MHT) codes.
D	WWF_LEVEL_II_MHT_CATEGORY	WWF level II major habitat type (MHT) names.

Data release file: Lookup_Table_5_ECOREGION_CODE.xlsx (.csv)^16^.

**Table 27:** PLANT FUNCTIONAL TYPE CODE (Lookup Table 6) field descriptions.

Column	Field Name	Description
A	PLANT_FUNCTIONAL_TYPE_CATEGORY_ABBREVIATION	Plant functional type (PFT) category abbreviations.
B	PLANT_FUNCTIONAL_TYPE_CATEGORY	Plant functional type (PFT) category names.

Data release file: Lookup_Table_6_PLANT_FUNCTIONAL_TYPE_CODE.xlsx (.csv)^16^.

## Methods

Below we describe the methods (adapted and updated from Strickland *et al*.^14^) used to create the data in each of the midden database tables. To the extent possible, we recorded midden data exactly as they were presented in their original source publications. However, in many cases we supplemented the published data with improved geographic location data and updated taxonomic classifications. In this version of the midden database (version 5.0), we have also calibrated the radiocarbon sample age data, added PFT data for midden taxa, and added ecoregion, climate, and bioclimate data for the midden sites.

Not all midden samples in the midden database have complete data. Table fields that contain “99999” values indicate that published data were not available or that we were unable to generate the appropriate information (e.g., sample sites without location data could not be assigned to an ecoregion). Blank table cells may indicate that published data were not available (e.g., missing location data) or blank cells may appear in unused fields such as notes or comments fields, fields with flags, and secondary data fields. The abbreviation “n/a” (i.e., not applicable) was used when a field was not applicable to the record represented by the table cell.

### Linked Tables 1–10

The 10 Linked data tables contain the midden macrofossil data fields displayed in Fig. 2. These data tables were used to create the relational database.

### Linked Table 1: REFERENCE

The REFERENCE table lists the 336 published sources, including journal articles, book chapters, theses, dissertations, government and private industry reports, and conference proceedings, containing midden data that have been entered into the midden database. Each source publication is represented by a unique reference number (REFNUM). Various data elements in the midden database, such as midden ages, location data, and taxon lists, are tied to source publications using REFNUMs. The midden database also contains unpublished data that were contributed by various researchers. Sometimes a midden sample had data available from a published source, and additional contributed but unpublished data were added to the sample record in the MIDDEN SAMPLE table (Linked Table 2) or the AGEC14 table (Linked Table 3). Some data are from unpublished sources only, or data related to a particular sample are known to exist, but they are not published, and permission has not been granted to include these data in this database. All unpublished data sources are represented in the MIDDEN SAMPLE table (Linked Table 2) and AGEC14 table (Linked Table 3) source reference fields by the last name of the investigator(s) followed by “unpublished”. Further information about existing unpublished data not included in this database may be available from the noted investigator. Full references for all published data sources can be found in the FULL_REFERENCE field. The REFERENCE table consists of the four fields listed in Table 5.

### Linked Table 2: MIDDEN SAMPLE

The MIDDEN SAMPLE table provides location information for 3,331 midden samples and contains 109 different fields (Table 6). Each midden sample is identified by a unique sample code (SAMCODE). Midden-sample site location information, including latitude, longitude, elevation (m), state or province, county or regional district, country, and general locality description (e.g., was the site located in a particular mountain range or stream valley), was recorded directly from the written reports describing the midden-sample locations. For some midden-sample sites, latitude and longitude, elevation, and general locality information were interpreted from site maps included in the written reports describing the midden samples or were estimated from topographic maps and these data are labelled as estimates in the source fields.

In some cases, there may be multiple reference sources containing details about a specific midden sample. The REFERENCE1 field almost always lists the primary published source for information about a particular midden sample. The primary source is typically the earliest publication of the midden data, which usually includes the plant macrofossil taxon list. If a taxon list is available for a midden, its source publication is assigned as REFERENCE1, and thus REFERENCE1 is always the source of the taxon list. Additional sources of new or republished information for a midden are listed as additional references in the fields REFERENCE2 to REFERENCE17. References are followed by their corresponding REFNUMs found in the fields REFNUM1 to REFNUM17. REFNUMs are defined in the REFERENCE table (Linked Table 1) where complete references are provided. Sometimes macrofossil taxon lists have been published multiple times in different sources. In these instances, we compared subsequent taxon lists and noted in the COMMENTS field any unexplained discrepancies or corrections made to the original list by the author(s). If identifications, taxonomic names, and/or quantifications changed in subsequent publications, the original published macrofossil data from REFERENCE1 were edited to reflect these changes.

There are 2,336 midden samples (approximately two-thirds of the samples) that have associated taxon lists in the midden database. A symbol in the MIDDEN SAMPLE table’s TAXON_LIST_CODE field indicates when a taxon list is available for the midden sample. These symbols are defined in the TAXON LIST CODE table (Lookup Table 4). The symbol “C” indicates that a complete published taxon list is available, and the symbol “P” indicates that only a partial taxon list is published and available. Sometimes midden analysts were looking for the presence of particular plant taxa, and only those taxa were reported. When a complete taxon list is published, we assume that all taxa in the midden sample were reported. The symbol “CU” indicates that a complete but unpublished taxon list exists, and the symbol “PU” indicates that a partial but unpublished taxon list exists, however these unpublished taxon lists are not included in the database because the author has not given permission to release these data. The TAXON_LIST_SOURCE field provides the REFNUM of the primary taxon list source publication and multiple sources may be listed if a taxon list has been updated in a subsequent publication.

Most midden studies carried out prior to 2000 CE provided midden-site location coordinates as degrees, minutes, and seconds of latitude and longitude. More recent studies use the Global Positioning System (GPS) for locating sites, and site coordinates are commonly published using decimal degrees or UTM (Universal Transverse Mercator) coordinates. We record all midden locations by their original published latitude and longitude coordinates, which may be recorded as degrees, minutes, and seconds, decimal degrees, or both. Midden-sample location latitudes and longitudes reported only in decimal degrees were converted to degrees, minutes, and seconds in order to populate latitude and longitude set 1 fields [i.e., LATDEG(1), LATMIN(1), LATSEC(1), LONGDEG(1), LONGMIN(1), LONGSEC(1)]. All midden-sample locations reported as degrees, minutes, and seconds from set 1 were converted to decimal degrees using TOPO! Software^20–22^ and recorded in the latitude and longitude decimal degree fields (LATDECDEGCON, LONGDECDEGCON). A midden’s location data, such as latitude and longitude coordinates and elevation values, may have been published in multiple sources. We recorded every occurrence of each location data element that we found in the scientific literature along with the corresponding publication source. Repeatedly published location data are not always consistent, and data values frequently change through time, sometimes because of improved knowledge or sometimes because of researcher errors. Recording all location data occurrences in the literature allows us to evaluate which data values are most consistent and therefore may be most reliable and accurate. Multiple fields can be found in the MIDDEN SAMPLE table showing up to five latitude and longitude coordinate value sets [e.g., LATDEG(1), LATMIN(1), LATSEC(1), LONGDEG(1), LONGMIN(1), LONGSEC(1) form one coordinate set] and up to five elevation values [e.g., ELEV(M)1], with their corresponding source publications [e.g., SOURCE(1) and SOURCE_EL(1)]. For latitude, longitude, and elevation, the first set of values (i.e., identified with a “1” in the variable name) are typically those that are published most frequently and consistently or are derived from the original publication describing the midden sample and we therefore consider these data most reliable. When multiple published location data exist for a sample, we suggest using the first set of latitude, longitude, and elevation values (i.e., those identified with a “1” in the variable name).

Accurate location and elevation data are very important when conducting paleoenvironmental reconstructions and modeling exercises. Original published location data were not always precise, especially for locations described before the availability of GPS technology, which tended to have very general location data. In the MIDDEN SAMPLE table, we provide improved midden-location data for some samples based on original published coordinates, maps, and site descriptions. Improved location data (latitude, longitude, and elevation) were created by plotting the original published latitude and longitude coordinates using the geographic mapping software TOPO!^20–22^. The mapped midden locations were then compared to the published descriptions to assess whether the published coordinates accurately matched the published written description and/or location map. We checked each location for consistency, and in some cases, the plotted locations did not match the published description. When the coordinates significantly differed from detailed published accounts, we moved the point location within TOPO! to a new location that we considered an improved and better representation of the midden latitude, longitude, and elevation. Improved location estimates are represented in the “best location” (BEST_LATDEG, BEST_LATMIN, BEST_LATSEC, BEST_LONGDEG, BEST_LONGMIN, BEST_LONGSEC) and “best elevation” fields [BEST_ELEV(M)]. The BEST_SOURCE and BEST_LOCALITY_NOTES fields describe the resources used to improve the location estimate and the decisions made when altering the original location. When utilizing midden data for paleoecological reconstruction or modeling exercises, we recommend using these improved “best” location data instead of the original published location data. Note that if the sample site was considered a sensitive location at the time the site data were added to the database (e.g., Bechan Cave, Hoopers Hollow, Sandblast Cave) the location data were generalized (i.e., the latitude and longitude do not represent the exact site location) and this adjustment is noted in the MIDDEN SAMPLE table fields [e.g., LOCALITY_NOTES, BEST_LOCALITY_NOTES, SOURCE(1)] for the site.

To assist database users, the USE_STATUS field contains a numeric code that describes the completeness of the age data and taxon list for each midden sample. A USE_STATUS value of 1 indicates that the age data and taxon list are complete for the midden sample. USE_STATUS codes not equal to 1 indicate that the age data and/or taxon list are not complete (see Table 6 USE_STATUS code descriptions).

### Linked Table 3: AGEC14

This table provides the radiocarbon (^14^C) ages for individual midden samples. It is linked to the MIDDEN SAMPLE table (Linked Table 2) by the sample code (SAMCODE) field. The radiocarbon age data include radiocarbon ^14^C dates with standard deviations (s.d.), laboratory numbers, material dated, and calibrated radiocarbon ages. The AGEC14 table consists of the 19 fields listed in Table 7.

**Fig. 3:**
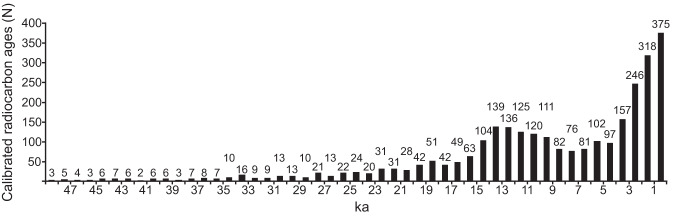
Histogram of the 2,859 calibrated radiocarbon ages (ka) in the midden database. The number above each bar indicates the number of midden-sample calibrated radiocarbon ages that fall within each 1-kyr age bin. Calibrated age data are from the CALIB704_CALENDAR_YR_BP_MEDIAN_PROBABILITY field in the AGEC14 table (Linked Table 3).

Radiocarbon ages were calibrated using CALIB (rev. 7.0.4)^23^ and the IntCal13.14c radiocarbon age calibration curve^24^, with the CALIB options for 2-sigma and Cal BP selected. This version of CALIB does not calibrate radiocarbon ages less than 71 ^14^C years or greater than 46,401 ^14^C years. Radiocarbon ages without reported standard deviations and infinite ages (for example, reported as greater than 30,000 years) were not calibrated. Infinite radiocarbon ages reported with a greater than sign (“>”) cannot be entered as a numeric value into the AGEC14 field, therefore for infinite ages, we dropped the greater-than sign and added one year to the age (for example, an age of >30,000 was input as 30,001), and an explanation of the change was added to the COMMENTS field. If a radiocarbon age is reported with a standard deviation where the positive value (“+”) is different from the negative value (“−”), we used the larger absolute value for the calibration. Middens reported or observed as modern but not dated are recorded in the database with a ^14^C age of “0” and a standard deviation of “99999” and these ages were not calibrated. Middens with ages reported as post 1950 with percent modern carbon are recorded in the database as modern with a ^14^C age of “0” and a standard deviation of “99999” and were not calibrated. Average ages created by investigators also were not calibrated. For radiocarbon ages that meet CALIB requirements, we used CALIB to generate the calendar year BP (before present) median probability, the absolute lower calendar age, the absolute upper calendar age, and the 95% (2-sigma) calendar age range. There are 2,859 midden samples for which we provide calibrated radiocarbon ages. The sample ages have a bimodal distribution, with large numbers of samples dated to the late Holocene and to the late Pleistocene (Fig. 3). Thompson *et al*.^25^ discussed potential reasons that the midden calibrated ages might display this bimodal distribution, including paleoclimatic effects, differences in midden preservation over time, and preferential sampling of particular time periods by researchers. Note that CALIB (rev. 7.0.4)^23^ used the IntCal13 curve for radiocarbon age calibration^24^. The more recent IntCal20 curve^26^ may produce different calibrated ages, particularly during the late Pleistocene^27^, and database users interested in this time period may want to use the IntCal20 curve to calibrate the database’s radiocarbon ages. Differences in the calibration curves can be seen by visually comparing the curves (e.g., Fig. 4 in Reimer *et al*.^26^).

In some cases, multiple subsamples from the same larger midden sample have been dated and such subsamples will have the same SAMCODE value in the AGEC14 table. If multiple ages on the same midden sample are within 2 standard deviations (2 sigma) of each other, we pooled the ages with CALIB. To calculate a single pooled age (PA), we used CALIB by first selecting from “Tools” and running: “Test Sample Significance”. If the results indicated that the samples were statistically the same at the 95% confidence level, we proceeded and created a pooled mean, by choosing: “Create Pooled Mean” and “Calibrate (GO)”. The output file “calout.csv” contained the pooled (averaged) ^14^C year and calendar year age. There were some instances when multiple radiocarbon ages were within 2 standard deviations of each other, however CALIB determined that the samples were not statistically the same. In these cases, we calculated a pooled mean regardless of the fact that the samples failed CALIB’s significance test. We ran the create pooled mean/calibrate calculation as above, but we note when, according to CALIB, the samples were not statistically the same age, and the pooled age (PA) is labelled as “PA(FST)” where “FST” stands for “Failed Significance Test”.

When multiple ages exist for a given midden sample, it may be confusing as to which age best represents the calendar age of the midden macrofossil assemblage. In the field called RECOMMENDED_AGE_OF_MIDDEN_ASSEMBLAGE we suggest a single age that best represents each sample/macrofossil assemblage by using a series of symbols [X, X1-PA, X1-PA(FST), X2]. When a midden sample has only a single age, that age is marked with an “X”, designating it as the recommended age of the midden. When multiple ages are listed and they are statistically the same, we recommend using a pooled age [PA or PA(FST)] as the primary preferred age to represent the sample/macrofossil assemblage. Pooled ages are designated with an “X1-PA” or “X1-PA(FST)” where “X1” indicates the primary preferred age. When multiple ages are present, instead of using a pooled age, a user may also choose one of the individual ages to represent the midden assemblage, such as an age on *Neotoma* spp. fecal pellets or an age on a specific plant taxon. We use the symbol “X2” to identify a secondary preferred age. When choosing a single age amongst multiple ages to represent an assemblage, rather than using a pooled age, the following rules apply: (1) when multiple uncalibrated radiocarbon age ranges overlap within 2 standard deviations, we recommend using the age with the smallest standard deviation and the corresponding calibrated age; (2) if the ages are reported as infinite, we recommend using the oldest age to represent the midden; (3) if the standard deviation is the same for multiple ages, we recommend using the oldest age; and (4) if one age is finite and one age is infinite, we recommend using the finite age. When recommending a preferred age to represent a midden assemblage, we provide justification for the decision within the RECOMMENDED_AGE_JUSTIFICATION field.

When a midden has multiple ages and they are not within two standard deviations of each other, it is not possible to determine which age would best represent the midden assemblage. In this case, we use a “?” in the RECOMMENDED_AGE_OF_MIDDEN_ASSEMBLAGE field to indicate that the age of the assemblage is uncertain, and we are unable to assign a single age. We occasionally use “X?” to designate a recommended age, which indicates that there is some uncertainty about the age due to issues such as mixed assemblages or confusing changes in interpretation by the author, therefore midden assemblages with ages marked with “X?” should be used with caution.

### Linked Table 4: MIDDEN TAXA

The MIDDEN TAXA table lists the 1,815 plant macrofossil taxa recorded from the midden samples. Each plant taxon in the MIDDEN TAXA table has a unique identifying variable number (VARNUM). The Latin botanical names for each taxon have generally been recorded in the VARNAME field in the same format as they were published by the midden macrofossil analysts. To maintain data consistency, because italics are not possible in .csv file formats, Latin genus and species names are not italicized in the database tables although use of italics is the correct style.

If the nomenclature or syntax of the taxon name used by the midden analyst did not conform to the rules of this database, we modified the name to match the database rules if the modification did not alter the meaning or level of uncertainty of the taxon identification. Where we have modified the nomenclature or syntax of a botanical name, the original name and any changes applied will be described in the NOTES field. The MIDDEN TAXA table consists of the 12 fields described in Table 8.

### Rules of nomenclatural synonymy

Botanical nomenclature is the formal system for naming plants, which is governed by the International Code of Botanical Nomenclature (ICBN)^28^. Plant taxa are often renamed when they are assigned to new families, genera, species, or infraspecific (varieties and subspecies) subdivisions of classification as our understanding of the inter-relationships of plant taxa changes. Consequently, taxonomic distinctions available to midden analysts decades ago frequently did not include many of the choices available today. The MIDDEN TAXA table VARNAME field lists the original Latin botanical names of plant macrofossil taxa as they were published by midden analysts. If the botanical nomenclature used by the analyst is no longer accepted as the taxon’s formal botanical name due to taxonomic changes, then the new currently accepted taxon name (synonym) is listed in the CURRENTLY_ACCEPTED_VARNAME field. Authorities and source references for old and new taxon names, as well as common names and family names are also included in the MIDDEN TAXA table.

Botanical nomenclature in the midden database generally follows the Integrated Taxonomic Information System (ITIS, https://www.itis.gov, accessed 2014–2019). Common names and family names also follow ITIS. All original plant macrofossil taxon names were cross-checked with ITIS as well as with the Tropicos database (http://www.tropicos.org, accessed 2014–2015) and the USDA NRCS PLANTS Database (https://plants.sc.egov.usda.gov, accessed 2015) to verify correct spelling, authority names, and status of acceptance of the botanical name. Through this cross-checking process, we determined that ITIS was the most complete, consistent, and reliable source dataset for verifying plant macrofossil botanical names. We therefore used ITIS as the main source for current botanical nomenclature. However, if ITIS did not provide sufficient information on current nomenclature, we used the following additional sources for nomenclature verification: Anderson^29,30^, Calflora (https://www.calflora.org/), The Gymnosperm Database (https://www.conifers.org/, accessed 2011), eFloras (http://www.efloras.org), Gentry^31^, Gleason & Cronquist^32^, Guzmán *et al*.^33^,Tropicos.org (http://www.tropicos.org), USDA NRCS PLANTS Database (https://plants.sc.egov.usda.gov), Webb & Starr^34^, and Wiggins^35^. The source reference used for verifying each taxon is listed in the NOMENCLATURAL_SOURCE field. Other nomenclatural resources consulted and mentioned in the NOTES field include: Bye^36^, de Laubenfels^37^, Farjon^38^, Felger^39^, Jepson eFlora (https://ucjeps.berkeley.edu/eflora/, accessed 2014), Kartesz^40^, McLaughlin^41^, Starr^42^, The Plant List^43^, Turner *et al*.^44^, USDA Agricultural Research Service National Plant Germplasm System (https://www.ars-grin.gov/npgs/, accessed 2015), Vanden Heuvel^45^, and Wilder *et al*.^46^.

When a plant species is transferred to a new genus and species, sometimes the old species name is not directly synonymous with one of the new infraspecific taxa. Thus, ITIS specifies the autonym of the new taxon as the accepted name for the old taxon. The autonym is a botanical name with repeated species epithet and infraspecific name. For example, the taxon *Ericameria viscidiflora* is no longer recognized as an accepted botanical name, and its new accepted name or synonym is the autonym *Chrysothamnus viscidiflorus* ssp. *viscidiflorus*. Autonyms are automatically established when an infraspecific taxon is described and validly published. The autonym has no authority, and it shares the type material and morphological description of the original described species. For autonyms, ITIS places the species rank authority after the trinomial (genus, species, and infraspecific) names instead of after the binomial (genus and species) names. This placement is confusing and makes it appear that autonyms in the ITIS dataset have authorities, which they do not.

In paleoecological studies, it is important to consider that a plant macrofossil taxon identified in a midden at the species rank, such as *Ericameria viscidiflora*, could potentially belong to any of the former subspecies of *E. viscidiflora* or their synonymous currently accepted subspecies of *Chrysothamnus viscidiflorus*. The midden analyst may have chosen not to identify the original *Ericameria viscidiflora* specimen to the infraspecific level or they were unable to do so. For macrofossil taxa such as *Ericameria viscidiflora*, for which the currently accepted name is an autonym (*Chrysothamnus viscidiflorus* ssp*. viscidiflorus*), it may be more useful to consider these taxa as synonymous with the species rank (*Chrysothamnus viscidiflorus*), which includes all of the species’ infraspecific taxa. A species includes all of its subordinate taxa, whereas the subspecies autonym excludes any other subspecies.

Examples of nomenclatural synonymy from the database (currently accepted names in bold font) include:

Family level synonymy

1. **Asteraceae** = Compositae
2. **Poaceae** = Gramineae

Genus level synonymy

1. ***Ambrosia dumosa*** = *Franseria dumosa*
2. ***Forsellesia*** **sp**. = *Glossopetalon* sp.

Species level synonymy

1. ***Larrea tridentata*** = *Larrea divaricata*
2. ***Lappula squarrosa*** = *Lappula fremontii*

### General rules of syntax

Syntax rules apply to the non-Latin parts of botanical names. In the identification of plant macrofossils, syntax helps to convey the degree of certainty of the taxon identification. The midden database syntactic rules generally follow those defined by Birks & Birks^47^, The North American Pollen Database Manual^48^, Sigovini *et al*.^49^, and Watts & Winter^50^. Examples of syntax commonly used in the identification of plant macrofossils from packrat middens are listed below along with descriptions of the syntax used in this midden database. The following syntax, “cf.”, and “type” rules text is from Strickland *et al*.^14^ (pp. 12–16), updated as needed for version 5.0 of the midden database.

1. Family level use of syntax rules:
  A. If a specimen (or specimens) can only be identified to the family level, the following syntax is often used.
    Family => Asteraceae*
    Family sp. => Asteraceae sp.
    *Use of the family name alone (Family => Asteraceae) is preferred. Data that do not conform to this syntax are modified. For example, Family sp. => Asteraceae sp. is changed to Family => Asteraceae.
  B. When multiple specimens are identified to the family level, and more than one morphological type is distinguished but the genera cannot or were not determined, the abbreviations “undiff.” (undifferentiated) or “spp.” (presence of multiple genera/species within a family) are commonly used and the abbreviation “undet.” (undetermined) is occasionally used.
    Family undiff. (genera or species are undifferentiated) => Asteraceae undiff.*
    Family spp. (multiple genera/species) => Asteraceae spp.
    Family undet. (undetermined) => Asteraceae undet.
    *This database uses Family undiff. rather than Family spp. or Family undet. Data that do not conform to this syntax have been modified.
  C. When a specimen(s) belongs to one of two similar families and assignment to a single family cannot be made, the following syntax is often used.
  Family/Family => Chenopodiaceae/Amaranthaceae*
  Family or Family => Chenopodiaceae or Amaranthaceae
  Family – Family => Chenopodiaceae – Amaranthaceae
  *If a distinction between two similar families cannot be made based on morphology alone, a slash should be used between multiple family names rather than using “or” or “–”. Data that do not conform to this syntax have been modified. Chenopodiaceae is no longer recognized as a separate family, and it is now synonymous with Amaranthaceae. However, Chenopodiaceae/Amaranthaceae was a common assignment previously used by midden analysts.
2. Genus and species level use of syntax rules:
  A. When the genus and species are known, the taxon should be written as:
    *Genus species* => *Quercus gambelii*
  B. When the genus is known and the species cannot be determined, the taxon should be written as:
    *Genus* sp. => *Quercus* sp.
  C. When multiple specimens belong to the same genus, but multiple species may be present, the following syntax is commonly used.
    *Genus* undiff. (undifferentiated) => *Quercus* undiff.*
    *Genus* spp. (multiple species) => *Quercus* spp.
    *The database uses *Genus* undiff. rather than *Genus* spp. Data that do not conform to this syntax have been modified.
  D. When a specimen(s) belongs to one of two similar genera, the following syntax is commonly used.
    *Genus*/*Genus* => Rumex/Polygonum*
    *Genus* or *Genus* => *Rumex* or *Polygonum*
    *Genus* – *Genus* => *Rumex* – *Polygonum*
    *If a distinction between two similar genera cannot be made based on morphology, a slash is used between multiple genus names, rather than “or” or “–”, and the abbreviation sp. (species) after the genus name is dropped. Data that do not conform to this syntax have been modified.
  E. If a distinction cannot be made between two species, the following syntax is often used.

*Genus species/Genus species* => Rhus aromatica/Rhus virens*

*Genus species/species* => *Rhus aromatica/virens*

*Genus species/G. species* => *Rhus aromatica/R. virens*

*The database uses *Genus species/Genus species* and data that do not conform to this syntax have been modified.

### *Placement of “cf.” rules*

If the family, genus, or species identification of a plant macrofossil is somewhat uncertain but the specimen resembles a specific family, genus, or species, the Latin abbreviation “cf.” (*confer*, compare) is used to indicate that a specimen has the form of a particular family, genus, or species. Use of “cf.” implies that there is some degree of uncertainty in the taxon identification that may be the result of a number of factors, including poor macrofossil preservation, inadequate reference material, or ill-defined morphology.

1. If the species identification is uncertain, “cf.” should be placed before the species name.
  *Genus* cf. *species => Quercus* cf. *gambelii*
  Abbreviating the genus name when using “cf.” is considered proper syntax, however this rule is not commonly followed by macrofossil analysts and thus in this database we do not abbreviate the genus when using “cf.”
  *Genus* cf. *G. species* => *Quercus* cf. *Q. gambelii* => *Quercus* cf. *gambelii*
2. If the genus or family identification is uncertain, “cf.” should be placed in front of the genus or family name:
  cf. *Genus* => cf. *Quercus*
  cf. Family => cf. Asteraceae
  Family cf. *Genus* => Asteraceae cf. *Brickellia*
3. This database does not use parentheses around “cf.” placed after the genus or family name such as:

*Genus* (cf.) => *Quercus* (cf.)

Family (cf.) => Asteraceae (cf.)

### Use of “type” rules

The term “type” is used when one macrofossil type is present in a midden-sample assemblage, and it could be assigned to three or more taxa. Analysts that use “type” in their specimen identification may or may not indicate which taxa are possible matches to the specimen. The term “type” should always be placed after the family, genus, or species name and preceded by a hyphen^48^. Taxon names not conforming to this syntax were modified. The following examples are accepted in the midden database.

1. Family-type => Asteraceae-type

2. *Genus*-type => *Quercus*-type

Rather than:   *Genus* type => *Quercus* type   

*Genus* (type) => *Quercus* (type)   

*Genus* s.l. (*sensu lato*, in the broad sense) => *Quercus* s.l.

3. *Genus* *species*-type => *Quercus* *gambelii*-type

4. *Genus*/*Genus*-type => *Avena*/*Festuca*-type

Rather than: *Genus*-type/*Genus*-type => *Avena*-type/*Festuca*-type

### Other syntax

Other acceptable syntax to be placed in front of the appropriate variety, subspecies, subgenus, or subfamily name include:

var. = variety

ssp. = subspecies

aff. = *affinis* in Latin, meaning the specimen has affinity with a known species

subgenus (not abbreviated)

subfamily (not abbreviated)

The use of the abbreviation indet. (*indeterminabilis*, indeterminate) indicates that the specimen(s) was too poorly preserved, incomplete, or damaged to be identified to a lower taxonomic level. The level of taxonomic uncertainty is specified as follows:

Family, genus indet. => Asteraceae, genus indet.

*Genus*, species indet. => *Quercus*, species indet.

### Hybrid taxa syntax

The syntax for officially named hybrid taxa is:

*Genus* X *species* => *Quercus* X *organensis*

The syntax for suspected hybrid taxa is:

*Genus species* X *Genus species* hybrid => *Pinus monophylla* X *Pinus edulis* hybrid

### Linked Table 5: MIDDEN TAXA PER SAMPLE

The MIDDEN TAXA PER SAMPLE table consists of the 5 fields listed in Table 9, including the variable numbers (VARNUM) identifying the plant macrofossil taxa that were collected from each midden sample. Plant taxon lists for each midden sample were input directly from the midden sample’s source references. When a midden sample’s plant taxon list was published in multiple sources, the taxon list from the most recent source was typically entered into the database and assumed to be the most accurate taxon list. An effort was made to record discrepancies among taxon lists published at different times in the NOTES field as well as in the COMMENTS field of the MIDDEN SAMPLE table (Linked Table 2). The original published relative-abundance or count data for each macrofossil taxon were entered in the MCOUNT field and the plant organ(s) identified were included in the TYPE field. Original published MCOUNTs may have been represented by symbols or numbers. In some cases, authors included a symbol to denote when a taxon was considered an older or younger contaminant. Some authors included suspected contaminants in the taxon list and marked them with a symbol such as a “?”, while other authors removed possible contaminant taxa from their taxon list. Since authors do not deal with contaminant taxa in a consistent way, we made a concerted effort to document any contaminants discussed by the author in the NOTES field of the MIDDEN TAXA PER SAMPLE table.

### Linked Table 6: CODE TRANSLATION

In addition to recording numbers of individual macrofossil specimens (raw counts), various counting methods have been used in packrat midden analyses to represent the relative abundance of plant macrofossil taxa collected from midden samples. Common counting schemes include the use of numeric codes or symbols to represent taxon relative abundance. The CODE TRANSLATION table describes the type of counting scheme used in the source publication for each midden-sample taxon list and consists of the 6 fields listed in Table 10.

To allow comparison of taxon count and relative-abundance data for midden samples that were analyzed using different counting methods, the counting scheme for each midden sample was converted to a standardized 0-1-2 relative-abundance scale (see rules in next subsection). Each source reference contributing a macrofossil assemblage to the database is listed in the REFERENCE field of the CODE TRANSLATION table, and the original counting scheme (TYPE_OF_COUNT field), with an explanation (EXPLANATION field) of how the scheme is translated to the 0-1-2 code, is associated with each source reference. The translation from original scheme to 0-1-2 code is uniquely defined for each source publication, therefore each midden-sample taxon list is linked to a single source publication. The source publication citation for each midden-sample taxon list in the database is found in the REFERENCE1 field of the MIDDEN SAMPLE table (Linked Table 2). Individual midden-sample codes are linked by the REFERENCE1 field to the REFERENCE field in the CODE TRANSLATION table, allowing the taxa in each sample to be translated according to the rules defined in the CODE TRANSLATION table.

#### Rules for Translating the MCOUNT Data into the 0-1-2 Presence-Absence Scale

The following rules text is from Strickland *et al*.^14^ (p. 18), updated as needed for version 5.0 of the midden database.

1. Counting schemes using symbols (e.g., *, X, +): A single symbol is represented in the 0-1-2 code by the code number “1” (rare) and two or more symbols (for example ** or ***) are represented by the code number “2” (present).
2. Numeric relative-abundance scales: A value of 1 is represented in the 0-1-2 code by the code number “1” (rare) and values ≥ 2 are represented by the code number “2” (present).
3. Percent abundance scales and raw counts: Values ≤ 5% or ≤ 5 specimens are represented by the code number “1” (rare) and values > 5% or > 5 specimens are represented by the code number “2” (present).
4. Macrofossil weights in grams: Values ≤ 0.003 grams are represented by the code number “1” (rare) and values > 0.003 grams are represented by the code number “2” (present).
5. Macrofossil abundance measured on a log_10_ of number of plant fragments/kg of washed matrix scale: Values ≤ 0.70 are represented by the code number “1” (rare) and values > 0.70 are represented by the code number “2” (present). The value 0.70 = log_10_ 5 macrofossils/kg.
6. If authors list contaminants in the taxon list, those taxa are assigned code number “9” and should not be used as part of the assemblage.
7. Sometimes we are unable to translate an original macrofossil count because the author has not sufficiently defined the symbols used in the counting scheme, and those taxa are assigned code number “7”. Note that if macrofossil counts for taxa from one publication were assigned code number “7” in the CODE TRANSLATION table (Linked Table 6), but an alternate published dataset with a more complete taxon list, and with no macrofossil counts assigned code number “7”, was chosen to represent the macrofossil assemblage, then no taxa with a code number of “7” will appear in the MCOUNT TRANSLATION table (Linked Table 7). This happens when the same data are published in multiple reports, however only one source can be designated as REFERENCE1 in the MIDDEN SAMPLE table. In this version of the midden database (version 5.0) no code number “7”s appear in the MCOUNT TRANSLATION table (Linked Table 7) because they are supplanted by data from the more complete source designated as REFERENCE1.
8. Absences are not frequently recorded by midden analysts. If a taxon list is complete (i.e., all midden taxa were identified) usually only taxa present are recorded (represented by code numbers “1” or “2”) and unlisted taxa are assumed absent. If a taxon list is complete and absences have been recorded, these absences are represented by the code number “0”. More commonly, if a taxon list is only partial (i.e., only certain midden taxa were identified and other potentially identifiable taxa were ignored), an author sometimes records the absence of certain taxa that were being sought out and these absences are also represented by code number “0”.

### Linked Table 7: MCOUNT TRANSLATION

This table is similar to the MIDDEN TAXA PER SAMPLE table (Linked Table 5). Both tables list the midden taxa from each sample assemblage by their variable numbers (VARNUM) and the original macrofossil count or relative-abundance value or symbol (MCOUNT) for each taxon is provided. The MCOUNT TRANSLATION table adds equivalent standard 0-1-2 codes for each macrofossil value or symbol in the MCOUNT field. The original macrofossil counts from the MIDDEN TAXA PER SAMPLE table (Linked Table 5) are translated into the standardized presence-absence 0-1-2 code using parameters defined in the CODE TRANSLATION table (Linked Table 6) and a make-table query. The MCOUNT TRANSLATION table consists of the four fields listed in Table 11.

### Linked Table 8: CLIMATE DATA

The CLIMATE DATA table contains climate and bioclimate data for the 2,929 midden-sample sites with original published location data or for which we estimated “best” location data. The CLIMATE DATA table consists of the 72 fields listed in Table 12. We interpolated CRU CL 2.0 (1961–1990 30-year mean^18^, data available at https://crudata.uea.ac.uk/cru/data/hrg/) mean monthly temperature (°C), precipitation (mm), possible sunshine (% of daylength), and mean diurnal temperature range (°C) data to each midden-sample site location (latitude and longitude) and elevation (m) using lapse-rate adjusted bilinear interpolation^51^. The same approach was used to interpolate World WeatherDisc absolute minimum and maximum temperature (1951–1980) data^52^ to the midden-sample site locations.

The interpolated monthly climate data (1961–1990 30-year mean) were used to calculate a set of bioclimatic variables (Table 12) for each midden site location using SPLASH^19^ (SPLASH code is available from https://bitbucket.org/labprentice/splash/src/master/). The monthly climate data were interpolated to a daily time step using a mean-preserving interpolation method^53,54^. Mean annual temperature (°C) was calculated as the month-length weighted average of monthly mean temperature. Chilling period was calculated as the number of days in the year with temperatures <5 °C. SPLASH was modified to include snow-water accounting: snowfall snow water equivalent (SWE) was calculated using a threshold air temperature of −1.0 °C at which all precipitation falls as snow and a threshold air temperature of 4.0 °C at which no precipitation falls as snow^55^. Actual evapotranspiration (AET) was calculated by SPLASH using a soil moisture capacity of 150 mm for all midden-sample site locations. The annual Priestley-Taylor coefficient (α) was calculated as AET divided by equilibrium evapotranspiration (EET), an evaporation-ratio variable (AETPET) was calculated as AET divided by potential evapotranspiration (PET), and the annual moisture index, MI, was calculated as annual total precipitation (ANNP) divided by PET (Table 12).

The midden-sample site latitudes, longitudes, and elevations used to estimate the climate data have various uncertainties associated with them that affect their locational accuracy. The BEST_LOCALITY_NOTES field in the MIDDEN SAMPLE table (Linked Table 2) describes many of the issues with these data and the steps that were taken to develop the “best” latitude, longitude, and elevation data for each site. Users of the climate data are encouraged to read through the BEST_LOCALITY_NOTES field information describing the adjustments to the site latitudes, longitudes, and elevations before using the climate data created for the midden-sample site locations.

Packrat middens are typically found in protected locations such as caves and crevices, and the climate and bioclimate data in the CLIMATE DATA table do not represent the climatic conditions in these protected locations. Packrat midden sites may be in canyons, cliff faces, and other topographic positions where topographic shading, slope, aspect, cold-air drainage, local moisture sources (e.g., streams), substrate type, etc., may modify local climatic conditions, which in turn may affect the taxonomic composition of the local vegetation within a packrat’s foraging area^56^. The climate and bioclimate data provided for the individual midden-sample sites may not capture these unique local conditions but instead should be considered estimates of the conditions in unprotected, open areas around the packrat midden sites.

### Linked Table 9: ECOREGION

All midden samples with either best location data (as estimated by us) or original published location data were plotted and classified according to ecoregion type. If specific latitude and longitude coordinates were not available, but the general location was known, the ecoregion could usually be deduced, however if the ecoregion could not be interpreted, we entered “99999”. Ecoregion classification follows the World Wildlife Fund ecoregions of North America scheme as described by Ricketts *et al*.^17^. We use the WWF major habitat types (MHTs) and ecoregion classifications, which we refer to as level II and level III categories, respectively. Ecoregions are grouped together to form MHTs, which “are not geographically defined units; rather, they refer to the dynamics of ecological systems and to the broad vegetative structures and patterns of species diversity that define them. In this way they are roughly equivalent to biomes.” (Ricketts *et al*.^17^, p. 13–14). An ecoregion is defined as “a relatively large area of land or water that contains a geographically distinct assemblage of natural communities. These communities (1) share a large majority of their species, dynamics, and environmental conditions and (2) function together effectively as a conservation unit at global and continental scales […]” (Ricketts *et al*.^17^, p. 7). Midden sites in this dataset occur within 6 different level II MHT categories and 24 level III ecoregion categories (Table 13). The ECOREGION table consists of the 5 fields listed in Table 14.

### Linked Table 10: PLANT FUNCTIONAL TYPE

Plant functional types (PFTs) group plant taxa by characteristics such as their stature, leaf form, phenology, and climatic adaptations^57–60^. These categories of plant functionality may be grouped together to form biomes, which provide the foundation for modeling past and future vegetation change^61–63^. Each macrofossil plant taxon in the packrat midden database has been assigned to one or more of 24 PFT categories (because individual taxa can belong to multiple PFTs). For each VARNUM in the PLANT FUNCTIONAL TYPE table (Table 15), PFT categories (table columns B-Y) contain either a value of “0” or “1” indicating the PFTs to which each taxon belongs. A “1” indicates that the midden taxon is assigned to the PFT category and a “0” indicates that the taxon is not assigned to the PFT category. The PLANT FUNCTIONAL TYPE table consists of the 27 fields listed in Table 15.

PFT categories were modified from those used to classify European pollen taxa for biome reconstruction^58^, and our PFT assignments and definitions are similar to those applied to pollen taxa in biome reconstruction studies for Canada, Beringia, and the eastern United States^64,65^. The PFT categories used here closely follow those used for midden taxa in Thompson & Anderson^59^ where 5 new PFTs (steppe shrub, woodland conifer, woodland shrub, desert shrub or succulent, and frost sensitive desert shrub or succulent) were created to accommodate plants from southwestern United States steppe, woodland, and desert plant communities. This expanded PFT classification successfully incorporates taxa common in packrat middens, however categories used for biomization are not always broad enough to capture the full climatic range of each midden taxon. For example, in the boreal climatic zone, PFTs do not include categories to represent broadleaved evergreens (*Arctostaphylos* sp.), succulents (Cactaceae such as *Opuntia fragilis*), or deciduous needleleaved plants (no representative taxa currently found in middens) which do occur there. However, these excluded PFT categories are not as characteristic of boreal climates as are needleleaved evergreen and broadleaved summergreen categories^58,66^. Some plant taxa found in the midden samples are difficult to assign to representative PFT categories, particularly taxa that may not be important components of PFTs, such as climatically widespread taxa (many forbs) or highly localized edaphic taxa (*Salix* spp. in riparian areas). In cases where there was not a good PFT match for a taxon, we assigned the taxon to a PFT in the climatic zone or biome where the taxon is most common.

PFT assignments were made based on descriptions of plant community ecology for individual plant species found in North American floras including Benson^67^, Benson & Darrow^68^, Carter^69^, Davis^70^, Great Plains Flora Association^71^, Harrington^72^, Hickman^73^, Hitchcock & Cronquist^74^, Kartesz^75^, Kearney & Peebles^76^, Munz & Keck^77^, Powell^78^, Powell *et al*.^79^, Roberts^80^, Taylor *et al*.^81^, Turner *et al*.^44^, and Welsh^82^. Additional online resources that were consulted are listed in Table 16. Table 17 describes the characteristics of plants included in each PFT category and lists representative midden taxa belonging to each category.

### Unlinked Tables 1–4

Four Unlinked data tables provide additional information that was compiled when creating the midden database and that users of the database may find useful.

### Unlinked Table 1: SYNONYMS

This table equates synonymous botanical names listed in the MIDDEN TAXA table (Linked Table 4) VARNAME field. Changes in botanical nomenclature may occur based on expert opinion and genetic studies that improve our understanding of the relationships between plant families, genera, and species. As a result, packrat midden analysts have used different botanical names to identify plant macrofossils belonging to the same plant taxon. For example, in the 1960s creosote bush was represented in North America by the taxon *Larrea divaricata* ssp. *tridentata*. During the 1970s, some scientists, including midden analysts, began to consider *Larrea divaricata* ssp. *tridentata* as a species distinct from *Larrea divaricata* and the new species was recognized as *Larrea tridentata*^83–86^. The new species distinction was controversial for decades, but genetic studies later verified that *Larrea tridentata* and *Larrea divaricata* were distinct species^87^ with *Larrea tridentata* only found in North America and *Larrea divaricata* only present in South America. Both names (*L. divaricata* and *L. tridentata*) have been used by midden analysts to identify creosote bush macrofossils, and both names appear separately in the VARNAME field of the MIDDEN TAXA table (Linked Table 4), even though they are synonymous names representing the same plant species. All synonymous names for a taxon [up to 8 names if there are multiple older names (VARNAME through SYNONYM_VARNAME8 fields)] are listed in the SYNONYMS table and are displayed across a single table row alongside their corresponding variable numbers (VARNUM through VARNUM8 fields). The SYNONYMS table consists of the 18 fields listed in Table 18.

Sometimes a plant taxon is identified with various degrees of certainty. A midden analyst may use syntax such as “cf.”, “-type”, “undiff.”, or two taxa separated by a slash to indicate various levels of certainty in an identification. We include names using such syntax as synonymous names, since there is potential for taxa identified with less certainty to be equivalent to the taxon of interest. Each database user should decide on the level of certainty of identification that is acceptable for their particular investigation. After each series of synonymous macrofossil taxon names, we also provide the currently accepted botanical name, however the macrofossil taxon may not be listed or identified by this name in the VARNAME field of the MIDDEN TAXA table (Linked Table 4). The currently accepted name is provided without any syntax indicating uncertainty (“cf.”, “-type”, “undiff.”, etc.) regardless of the syntax of the original botanical name used by the midden analyst.

If a botanical name has been revised and a species has been transferred to a lower rank of ssp. or var. under a new species (for example, the accepted name for *Andropogon hallii* is now *Andropogon gerardii* ssp*. hallii*), we only equate the old name (*Andropogon hallii*) with the new subspecies (*Andropogon gerardii* ssp. *hallii*) and we do not equate *A. hallii* with the new species (*A. gerardii*), even though *A. gerardii* appears in the MIDDEN TAXA table (Linked Table 4). It is possible that macrofossils identified as *A. gerardii* could include plants now known as *A. gerardii* ssp. *hallii*, as well as other subspecies, because a species is the sum of its infraspecific taxa. However, it is unknown which varieties the midden analyst considered in the *A. gerardii* identification. Depending on what point in time the identification was made, the analyst may have considered *A. gerardii* ssp. *hallii* an infraspecific taxon of *A. gerardii* or considered it a separate species (*A. hallii*).

Sometimes when a taxon is moved to a new genus or species, its accepted botanical name is the autonym of the new taxon, for example, the accepted name for *Condalia lycioides* is now *Ziziphus obtusifolia* var. *obtusifolia*. Considering that the macrofossil *C. lycioides* could potentially belong to a former variety of *C. lycioides* or a new variety of *Ziziphus obtusifolia*, we treat the old name *C. lycioides* as synonymous with both the autonym (*Ziziphus obtusifolia* var. *obtusifolia*) and the new species rank name (*Ziziphus obtusifolia*) if either name appears in the MIDDEN TAXA table (Linked Table 4). A note is included in the SYNONYMS table NOTES field to indicate when an autonym is the accepted botanical name, however it may be useful to also recognize the species-rank taxon, which includes all the species’ infraspecific taxa, as synonymous.

### Unlinked Table 2: MIDDEN RELATED PUBLICATIONS WITH NO SIGNIFICANT DATA

This table lists selected references published from 1875 to 2019 that were assessed for midden plant macrofossil data. These publications did not contain significant midden data and no data from these sources were included in the database. The MIDDEN RELATED PUBLICATIONS WITH NO SIGNIFICANT DATA table consists of the three fields listed in Table 19.

### Unlinked Table 3: MIDDEN RELATED ABSTRACTS WITH NO SIGNIFICANT DATA

This table lists references for a selection of oral and poster presentation abstracts published in conference proceedings (1974 to 2016) that are related to midden studies. These abstracts did not contain significant midden plant macrofossil data and no data from these sources were included in the database. The MIDDEN RELATED ABSTRACTS WITH NO SIGNIFICANT DATA table consists of the three fields listed in Table 20.

### Unlinked Table 4: MIDDEN PUBLICATIONS OUTSIDE NORTH AMERICA

This table lists a selection of references published from 1983 to 2004 for midden studies located outside of North America. Midden data available in this database are derived only from sites located in North America. However, as data were collected for North America, we kept a list of midden publications encountered that represented other areas. No data from these sources were included in the midden database and the list of these sources (Unlinked Table 4) is not meant to be a complete bibliography of midden publications for sites outside of North America. The MIDDEN PUBLICATIONS OUTSIDE NORTH AMERICA table consists of the three fields listed in Table 21.

### Lookup Tables 1–6

The six Lookup tables provide definitions of the various codes that are used in Linked Tables 2, 3, 6, 7, 9, 10 (Tables 6, 7, 10, 11, 14, 15).

### Lookup Table 1: USE STATUS CODE

Use status codes are used to indicate the completeness of data available for each midden sample. Detailed definitions were previously described in the MIDDEN SAMPLE table (Linked Table 2) description. The USE STATUS CODE table consists of the two fields listed in Table 22.

### Lookup Table 2: 0-1-2 CODE

This table defines the numeric codes used in the midden taxon 0-1-2 presence-absence scale. Relative-abundance or count data for each plant macrofossil taxon in a midden sample in the MIDDEN TAXA PER SAMPLE table (Linked Table 5, MCOUNT field) were translated into 0-1-2 code values using the code definitions in the CODE TRANSLATION table (Linked Table 6). The 0-1-2 CODE table consists of the two fields listed in Table 23.

### Lookup Table 3: RECOMMENDED AGE CODE

This table defines the codes used in the AGEC14 table to indicate ages recommended to represent each midden sample/assemblage. Detailed definitions were previously described in the AGEC14 table (Linked Table 3) description. The RECOMMENDED AGE CODE table consists of the two fields listed in Table 24.

### Lookup Table 4: TAXON LIST CODE

This table defines the taxon list codes that indicate whether a taxon list is available for a midden sample, if the taxon list is complete or partial, and whether the taxon list is published or unpublished. Detailed taxon list code definitions were previously described in the MIDDEN SAMPLE table (Linked Table 2) description. The TAXON LIST CODE table consists of the two fields listed in Table 25.

### Lookup Table 5: ECOREGION CODE

This table defines abbreviations used in the ECOREGION table (Linked Table 9) to represent 6 WWF level II major habitat types (MHTs) and 24 WWF level III ecoregions. Detailed definitions can be found in the ECOREGION table (Linked Table 9) description. The ECOREGION CODE table consists of the four fields listed in Table 26.

### Lookup Table 6: PLANT FUNCTIONAL TYPE CODE

Plant functional type codes were previously defined in the PLANT FUNCTIONAL TYPE table (Linked Table 10) description. The PLANT FUNCTIONAL TYPE CODE table consists of the two fields listed in Table 27.

## Data Records

The data described above are part of the USGS North American Packrat Midden Database version 5.0 data release^16^ and are available at 10.5066/P91UOARW. The Linked, Unlinked, and Lookup tables are provided within a relational database (.mdb) and are also provided separately as tabular (.xlsx) and comma-separated values (.csv) files^16^. Tables in the relational database are linked by simple joins connecting common fields containing unique values (Fig. 2). Each individual publication, midden sample, and plant macrofossil taxon recorded in the database is distinguished by a unique code. Publications are represented by reference numbers (REFNUM), midden samples by sample codes (SAMCODE), and macrofossil plant taxa by variable numbers (VARNUM).

An earlier version of the database (version 4.0, June 2016)^15^ can be accessed at https://geochange.er.usgs.gov/midden/. This online version of the database contains data for only 3,205 midden samples and does not contain the PFT and ecoregion assignments, climate and bioclimate data, recommended age data, or calibrated age data described in this publication. It allows the user to use a web browser to query the original published data and standardized data in the midden database by publication (author and date of publication), taxon, geographic area (state and country), locality name, latitude, longitude, and ^14^C age.

The relational database filename in the Strickland *et al*.^16^ midden database data release is: Strickland_and_others_2022_USGS_packrat_midden_Access_database_version_5.0.mdb

The Linked, Unlinked, and Lookup table .xslx (.csv) filenames in the Strickland *et al*.^16^ midden database data release are:

Linked_Table_1_REFERENCE.xlsx (.csv)

Linked_Table_2_MIDDEN_SAMPLE.xlsx (.csv)

Linked_Table_3_AGEC14.xlsx (.csv)

Linked_Table_4_MIDDEN_TAXA.xlsx (.csv)

Linked_Table_5_MIDDEN_TAXA_PER_SAMPLE.xlsx (.csv)

Linked_Table_6_CODE_TRANSLATION.xlsx (.csv)

Linked_Table_7_MCOUNT_TRANSLATION.xlsx (.csv)

Linked_Table_8_CLIMATE_DATA.xlsx (.csv)

Linked_Table_9_ECOREGION.xlsx (.csv)

Linked_Table_10_PLANT_FUNCTIONAL_TYPE.xlsx (.csv)

Unlinked_Table_1_SYNONYMS.xlsx (.csv)

Unlinked_Table_2_MIDDEN_RELATED_PUBLICATIONS_WITH_NO_SIGNIFICANT_DATA.xlsx (.csv)

Unlinked_Table_3_MIDDEN_RELATED_ABSTRACTS_WITH_NO_SIGNIFICANT_DATA.xlsx (.csv)

Unlinked_Table_4_MIDDEN_PUBLICATIONS_OUTSIDE_NORTH_AMERICA.xlsx (.csv)

Lookup_Table_1_USE_STATUS_CODE.xlsx (.csv)

Lookup_Table_2_0_1_2_CODE.xlsx (.csv)

Lookup_Table_3_RECOMMENDED_AGE_CODE.xlsx (.csv)

Lookup_Table_4_TAXON_LIST_CODE.xlsx (.csv)

Lookup_Table_5_ECOREGION_CODE.xlsx (.csv)

Lookup_Table_6_PLANT_FUNCTIONAL_TYPE_CODE.xlsx (.csv)

## Technical Validation

Data in the midden database have been evaluated and reviewed starting with the first release of the database in 1998. For this version of the midden database (version 5.0), data validation steps are described above for each data table and are summarized here. Midden-sample site location (latitude and longitude) and elevation data were recorded from each publication that provided this information for each midden site. Data for individual samples were often repeatedly reported in multiple source references allowing us to compare reported location and elevation data and identify data errors that may have propagated from one publication to the next. Midden site location and elevation data were also validated against TOPO! Software^20–22^ latitude, longitude, and elevation data, and adjustments were made to the midden-sample location data as described in the Methods section. Geographic names (e.g., the names of mountain ranges) were checked in the Geographic Names Information System (GNIS; https://www.usgs.gov/us-board-on-geographic-names/domestic-names) for locations in the United States and the GEOnet Names Server (GNS; https://www.nga.mil/resources/US_Board_on_Geographic_Names_.html) for locations in Canada and Mexico.

Changes in identification of a midden sample’s taxa or the quantification of macrofossil taxa from one publication to the next were noted. Taxon list discrepancies among publications describing the same midden sample were also noted and the recorded plant taxon names were cross-checked with ITIS (https://www.itis.gov, accessed 2014–2019), Tropicos (http://www.tropicos.org, accessed 2014–2015), and USDA NRCS PLANTS Database (https://plants.sc.egov.usda.gov, accessed 2015) databases and other published data sources.

Calibrated radiocarbon ages were plotted to check for data outliers (Fig. 3). Climate and bioclimate data were also checked for outliers. Names of geologic time periods used in the midden database conform to the usage approved by the USGS Geologic Names Committee^88^.

## Data Availability

Bioclimatic variables were calculated using SPLASH^19^. SPLASH code is available from bitbucket (https://bitbucket.org/labprentice/splash/src/master/).
